# Strong connections of locally clustered neurons in the upper-layer cortex underlie schizophrenia-related auditory deviance detection

**DOI:** 10.1126/sciadv.aeb4666

**Published:** 2026-08-14

**Authors:** Shunsuke Mizutani, Yasuhiro Go, Atsu Aiba, Kiyoto Kasai, Shigeo Okabe

**Affiliations:** ^1^ Department of Cellular Neurobiology, Graduate School of Medicine, The University of Tokyo, Tokyo 113-0033, Japan.; ^2^ Graduate School of Information Science, University of Hyogo, Kobe 650-0047, Japan.; ^3^ National Institute for Physiological Sciences (NIPS), National Institutes of Natural Sciences, Okazaki 444-8585, Japan.; ^4^ Exploratory Research Center on Life and Living Systems (ExCELLS), National Institutes of Natural Sciences, Okazaki 444-8585, Japan.; ^5^ Laboratory of Animal Resources, Center for Disease Biology and Integrated Medicine, Graduate School of Medicine, The University of Tokyo, Tokyo 113-0033, Japan.; ^6^ Department of Neuropsychiatry, Graduate School of Medicine, The University of Tokyo, Tokyo 113-8655, Japan.; ^7^ The International Research Center for Neurointelligence (IRCN), Institutes for Advanced Study (UTIAS), The University of Tokyo, Tokyo 113-0033, Japan.; ^8^ Laboratory for Imaging Neural Dynamics, RIKEN Center for Brain Science, Saitama 351-0198, Japan.

## Abstract

The detection of unexpected sounds, or auditory deviance detection, is thought to be achieved by comparing prediction signals and sensory inputs in the auditory cortex. This process is markedly impaired in patients with schizophrenia. However, the local circuit properties underlying deviance detection, together with their dysfunction in schizophrenia-related conditions, remain poorly understood. We used two-photon calcium imaging to monitor the population activity of cortical neurons during auditory deviance detection. The deviance-detecting component was predominant in layer 2/3 neurons of the higher-order auditory cortex. The deviance-detecting cells were spatially clustered, exhibited robust synchronization, and constitutively expressed immediate-early genes. In the Del(1.5 megabase)/+ mouse model of 22q11.2 deletion syndrome, a model of schizophrenia, deviance-detecting activity in the upper-layer network was disrupted, accompanied by dysregulated gene expression and selective loss of dendritic spines in immediate-early gene–positive neurons. Our findings support the hypothesis that ensemble activation of this neuronal subpopulation amplifies unexpected inputs, driving error signals that propagate to higher-order cortical areas.

## INTRODUCTION

The detection of unexpected changes in auditory stimuli serves a crucial function in animal perception because these deviant stimuli frequently contain valuable information for guiding subsequent behavior. According to the predictive coding hypothesis, the detection of deviant sounds in the auditory cortex (AC) is achieved by comparing two types of signals, top-down prediction signals and bottom-up sensory inputs, leading to the generation of prediction error signals that propagate toward higher cortical areas (*1*). A well-known support for the presence of prediction error signals in humans is mismatch negativity (MMN), an event-related potential that represents the difference in responses to frequent repetitive and rare deviant stimuli (*2*–*4*).

MMN is localized to the AC and is known to be preattentive, i.e., resistant to the manipulation of attention and consciousness (*5*). MMN, initially identified in humans, has been shown to be present in multiple species, including rodents, cats, and monkeys (*6*). Ulanovsky *et al.* (*7*) first reported single-neuron responses in the cat primary AC (A1) with a controlled stimulus paradigm to evoke MMN-like responses. They found strong stimulus-specific adaptation (SSA) in A1. Subsequent research on single-cell activity in the AC revealed a complex relationship between two dynamic components: SSA, which suppresses repetitive signals, and pure error signals, which are directly evoked by infrequent stimuli (*8*). Only under a few experimental conditions, the contributions of SSA and pure error signals on MMN-like responses were evaluated independently. For example, a recent study identified the rostral parabelt of the higher AC in marmosets as a source of pure error signals in response to deviant tones (*9*). Another challenge in the field is the amplification mechanism of error signals along the auditory pathway. A systematic evaluation of prediction error signals in the inferior colliculus, medial geniculate body, and AC reported an increase in error signals from the brainstem to cortex (*10*). A recent study proposed an intrinsic amplification mechanism within the AC (*11*). Furthermore, error signals were prominent in the nonlemniscal division of the AC, suggesting the contribution of long-range interactions of the nonlemniscal AC to either high-level cortical areas or subcortical structures (*12*). Although several models have been proposed for robust error signals in AC, the local circuit-level mechanism remains unknown.

In patients with schizophrenia, a substantial reduction in MMN amplitude has been observed, with substantial effect sizes (*13*–*15*). In addition, reduced MMN amplitude has been correlated with reductions in the gray matter volume of the AC (*16*, *17*). The deviance detection component of MMN, which is proposed to correspond to pure error signals in animal studies, is thought to provide insights into altered information processing in patients with schizophrenia (*18*). Specifically, prediction error mechanisms offer an attractive framework for explaining unusual beliefs, the most severe form of which is the delusions of patients with schizophrenia because these beliefs are associated with disturbed interplay between sensory evidence and false predictions. Therefore, studies on the underpinnings of MMN in the AC will contribute to clarifying the pathophysiology of auditory information processing in patients with schizophrenia.

A critical step toward translating MMN-related findings into mechanistic insight is the availability of animal models that faithfully recapitulate human pathology. To this end, we focused on 22q11.2 deletion syndrome, a condition that substantially increases the risk of a spectrum of neurodevelopmental disorders, including schizophrenia (*19*, *20*). Notably, carriers of the 22q11.2 deletion exhibit reduced MMN during adolescence (*21*, *22*), highlighting a clinically relevant and developmentally emerging deficit in deviance detection. To investigate the biological underpinnings of this deficit, we used the Del(1.5 Mb)/+ model, in which the syntenic genomic region is deleted in mice.

Human MMN studies have established the sophisticated tone presentation scheme that is essential for isolating deviance detection from SSA. This protocol has also been adapted for use in animal studies to identify the local circuit components underlying deviance detection. However, despite their importance, these deviance detection paradigms have not been applied to animal models of schizophrenia, leaving a critical gap between clinical observations and circuit-level mechanisms. To address this gap, it is necessary to record calcium responses from a large population of neurons in the AC of schizophrenia models, such as the Del(1.5 Mb)/+, using a tone presentation design to isolate deviance detection. Single-cell imaging in vivo provides rich information about local circuit activity, contributing to a comprehensive understanding of circuit impairments.

In this study, we successfully imaged calcium responses from a large number of neurons in response to tones designed to isolate deviance detection. Using this approach, we identified a neuronal population in the upper layer of higher-order AC with distinct properties, which we designated deviance-detecting (DD) neurons, characterized by spatial clustering, local synchrony, and elevated expression of immediate-early genes (IEGs). The DD neuron population in Del(1.5 Mb)/+ mice was reduced in number and showed markedly impaired functional and structural connectivity, with altered gene expression. Together, our findings demonstrate that functionally connected clusters of upper-layer neurons are critical for auditory deviance detection and are strongly affected by schizophrenia-related conditions. This discovery provides a mechanistic framework for interpreting MMN deficits in patients and links a clinically observed phenotype to specific circuit dysfunction.

## RESULTS

### Auditory response of subcortical and cortical circuits in Del(1.5 Mb)/+ mice

**Fig. 1.**
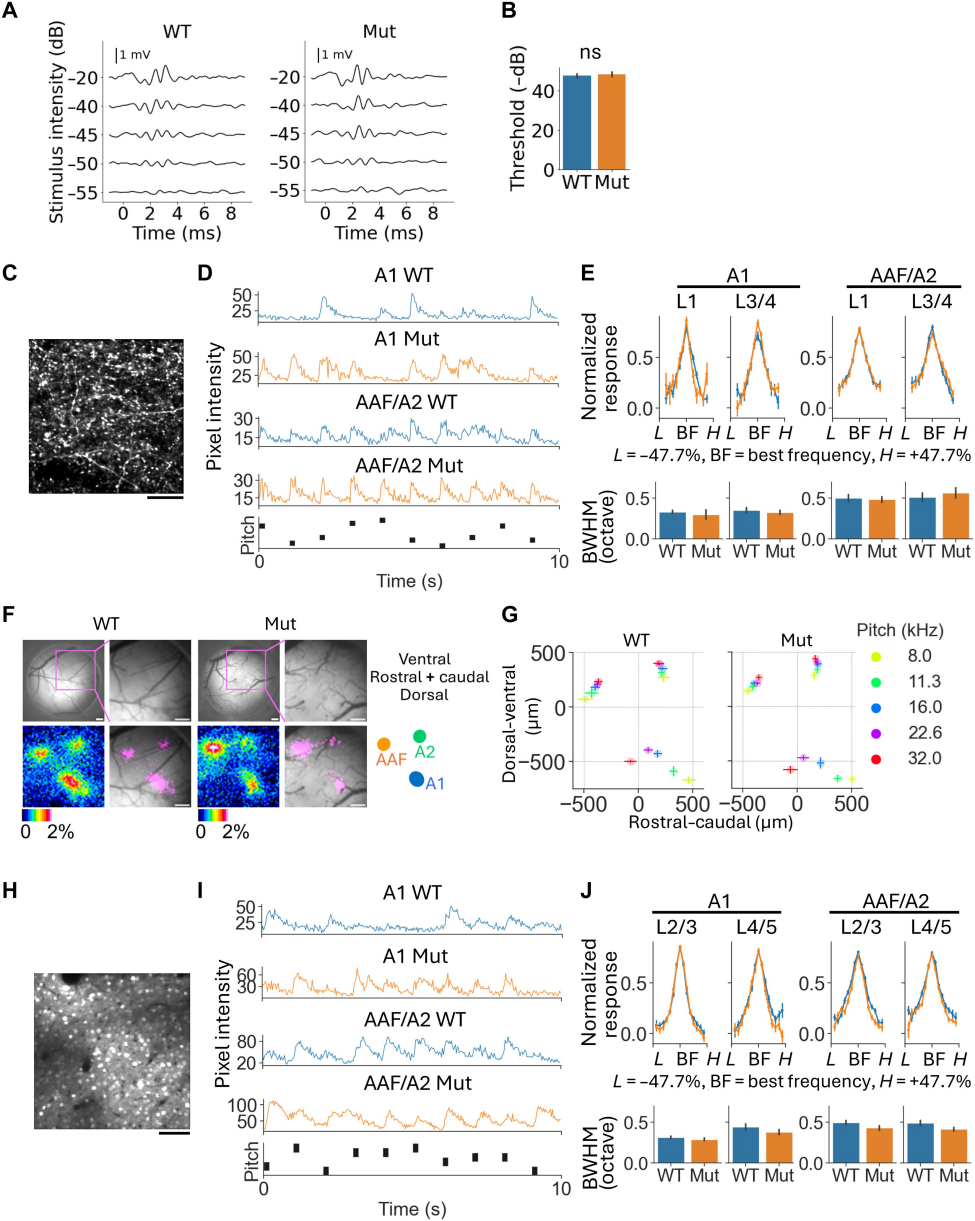
Auditory response of subcortical and cortical circuits in Del(1.5 Mb)/+ mice. (**A** and **B**) Representative auditory brainstem responses at different intensities (A) and averaged response thresholds in wild-type (WT; *n* = 6) and mutant (Mut, *n* = 6) mice (B). (**C** to **E**) Two-photon calcium imaging of thalamocortical axonal boutons. (C) Time-averaged GCaMP6f fluorescence in layer 1 (L1) of A1. Scale bar, 30 μm. (D) Single bouton responses to frequency-randomized pure tones. (E) Frequency profiles of bouton responses (top) and their bandwidth at half maximum (BWHM; bottom). There were no statistically significant interactions between genotype and cortical region (*P* = 0.4184), between genotype and layer (*P* = 0.6422), or among genotype, region, and layer (*P* = 0.4744). (**F** and **G**) Tonotopy analysis using wide-field imaging. (F) Mean responses to a pure tone (bottom left) indicate the positions of A1, AAF, and A2, with overlay to vascular pattern (bottom right), detected by time-averaging images (top left and top right corresponding to lower and higher magnifications). Scale bars, 300 μm. (G) Tonotopic maps showing tone-response means and standard error of measurement (SEM) by positions and sizes of the crosses. WT, *n* = 6; Mut, *n* = 6. (**H**) Time-averaged image of GCaMP6f-expressing neurons in L2/3 of the AC. Scale bar, 300 μm. (**I**) Single-neuron calcium responses to pure tones with random frequency. The pitch sequence is shown at the bottom. (**J**) Frequency profiles of neuronal responses (top) and their BWHM (bottom). There were no statistically significant interactions between genotype and cortical region (*P* = 0.7510), between genotype and layer (*P* = 0.4043), or among genotype, region, and layer (*P* = 0.6309). For the sample numbers, see Supplementary Data for Statistical Analysis. Comparison between groups were performed using Student’s *t* test. ns, not significant.

The presence of hearing impairment in mouse models mimicking the human 1.5-Mb deletion in the patients with 22q11.2 deletion syndrome has been previously reported, which compromised the evaluation of auditory information processing in the sensory cortex (*23*). For example, Df1/+ mice on the C57BL/6 genetic background showed peripheral hearing impairment associated with middle ear inflammation (*24*). We confirmed similar hearing deficits in Del(1.5 Mb)/+ mice on a C57BL/6N genetic background and found that this hearing impairment could be rescued by generating F1 hybrids from a cross between Del(1.5 Mb)/+ mice on a C57BL/6N background and mice of the CBA/CaOlaHsd strain. The advantages of using F1 hybrids between C57BL/6N and CBA/CaOlaHsd strains are their genetic homogeneity and high performance, which is likely attributable to heterosis. In particular, enhanced immunity to diseases in the F1 hybrids is likely involved in the prevention of middle ear inflammation in Del(1.5 Mb)/+ mice. No significant differences were observed in the auditory brainstem response thresholds of Del(1.5 Mb)/+ and control mice on the F1 hybrid of C57BL/6N and CBA/CaOlaHsd strains (Fig. 1, A and B), indicating the intact hearing ability of Del(1.5 Mb)/+ F1 hybrid mice and their usefulness in the analysis of deviance detection mechanisms in the AC.

Next, we evaluated the functions of thalamocortical projections using recombinant adeno-associated virus (AAV)–mediated expression of GCaMP6f in the medial geniculate body and subsequent in vivo two-photon imaging of thalamocortical axons in the AC (Fig. 1C). Control and Del(1.5 Mb)/+ mice showed similar calcium responses of single boutons to sound stimuli with different frequencies, further suggesting intact function of the subcortical auditory pathway (Fig. 1, D and E).

The auditory oddball paradigm with frequency alteration is based on the ability of subjects to discriminate between differences in frequency. To confirm the intact response of the AC to tones with different frequencies, we first performed wide-field single-photon imaging of pyramidal neurons expressing GCaMP6f using AAV over the entire auditory area (*25*). To obtain a precise cortical map of tonotopic organization, we recorded responses to pure tones of different frequencies. Through a large cranial window (diameter, 2.8 to 3.0 mm), the three subregions of the mouse AC, namely, A1, anterior auditory field (AAF), and secondary AC (A2), could be reliably identified on the basis of their tonotopic organization.

From the calcium responses to different frequencies, we created the best frequency maps for each mouse (Fig. 1, F and G). Although slight differences existed in the gradient and orientation, the overall features of the maps were preserved and similar between control and Del(1.5 Mb)/+ mice. Next, we performed two-photon imaging of calcium responses in single neurons to various frequencies in the three subareas of the AC: A1, AAF, and A2 (Fig. 1, H and I). Consistent with previous reports (*25*), frequency tuning of single neurons was sharper in A1 than in AAF and A2 (Fig. 1J); specifically, the bandwidth at half maximum (BWHM) of layer 2/3 (L2/3) neurons was significantly smaller in A1 compared to AAF/A2 in both wild-type (WT; *P* < 0.0001) and Del(1.5 Mb)/+ mice (*P* < 0.0001). The overall responses of the AC and single-neuron responses in Del(1.5 Mb)/+ mice indicated that the ability of this mouse model to discriminate different tone frequencies was not impaired and did not compromise the results of deviance detection using the oddball paradigm with altering frequency.

### Dysfunction of deviance detection in AAF/A2 of Del(1.5 Mb)/+ mice

**Fig. 2.**
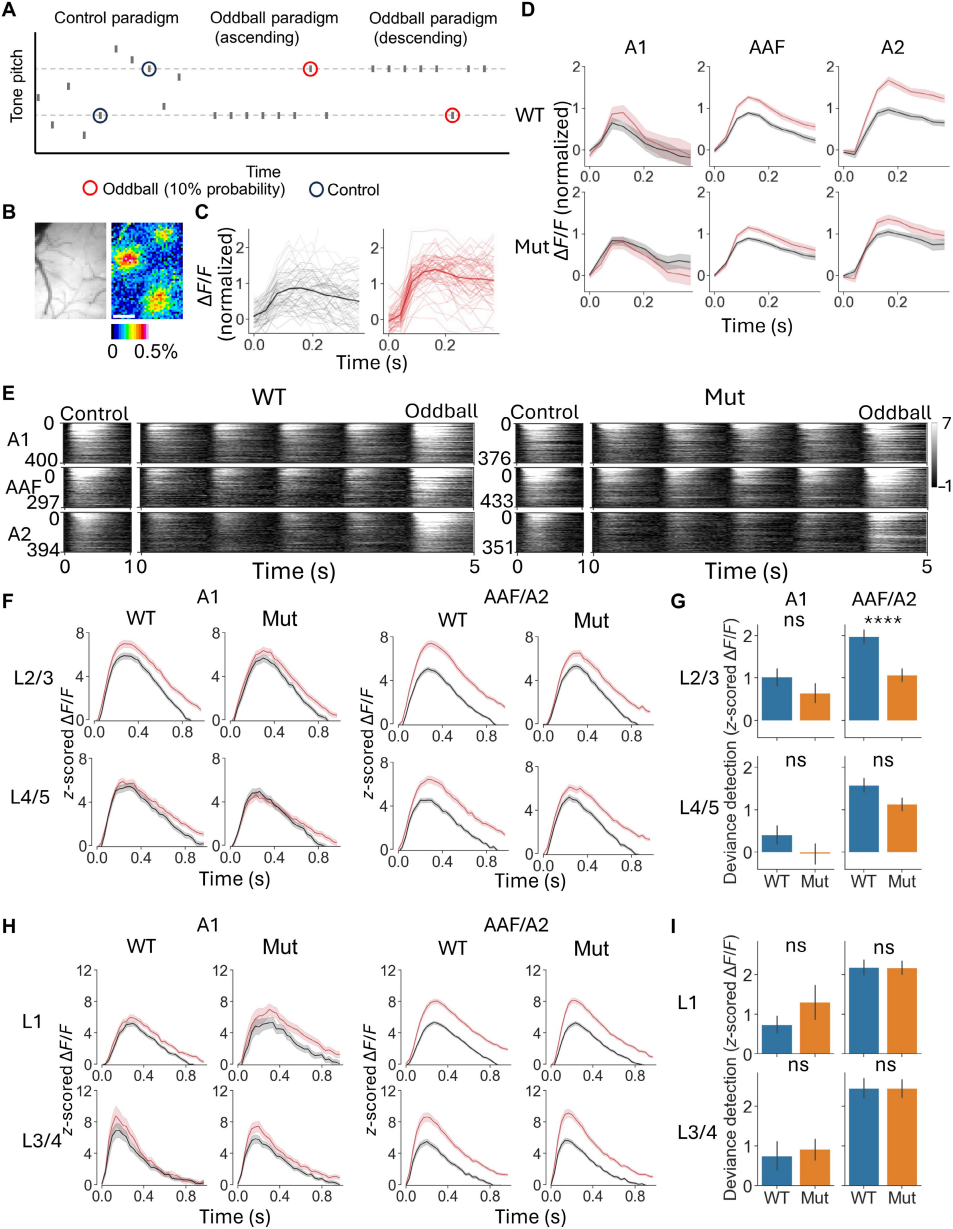
Dysfunction of deviance detection in AAF/A2 of Del(1.5 Mb)/+ mice. (**A**) Task design for deviance detection, consisting of pseudo-random pitch stimuli and ascending and descending oddball blocks with 10% rare stimuli. Comparisons were made between responses to tones with identical pitches (marked by circles). (**B** to **D**) (B) Wide-field imaging of RiboL1-jGCaMP8m-expressing AC neurons in WT mice. Mean response to pure tone stimulus illustrates AC subregions (right), with vascular pattern visualized by averaging all frames (left). Scale bar, 300 μm. (C) Representative A2 responses to the oddball (left) and control (right) stimulus in a WT mouse, with average of 60 trials (bold lines) and individual trials (thin lines). (D) Enhanced response to an oddball stimulus. The mean normalized responses with SEM (shaded areas) are plotted for the oddball (red) and control (black) responses. (**E** to **G**) (E) Two-photon imaging of single-neuron responses, presented as *z*-scores of calcium responses for control (1 s) and oddball (5 s) sessions. Deviant tones were presented in the last 1 s in the oddball paradigm. (F) Population average of single neurons responding to oddball and control stimuli. The shaded regions represent SEM. (G) Mean calcium response from 0 to 667 ms after the onset of the deviant tone. Statistical significance for the impairment in AAF/A2 L2/3 was confirmed by a two-way ANOVA (genotype × region/layer interaction) [*F*(1, 2253) = 4.43, *P* = 0.035], followed by a post hoc Bonferroni-corrected *t* test. (**H** and **I**), (H) Population-averaged responses from thalamocortical axonal boutons. Oddball (red) and control (black) responses with shaded areas indicating SEM. (I) Mean calcium response from 0 to 667 ms after the onset of the deviant tone in thalamocortical axonal boutons. ****P < 0.0001. For the sample numbers, see Supplementary Data for Statistical Analysis.

The first objective of this study was to identify DD activity in the mouse AC in response to an auditory oddball paradigm that mimics the protocol applied to patients with schizophrenia. An appropriate design for the auditory oddball paradigm is required to separate deviance detection from adaptation. Among the various forms of auditory oddball paradigms used in human MMN studies, we selected a protocol that has been shown to isolate pure deviance detection signals and is applicable to rodents (Fig. 2A). In this protocol, the difference in response between the oddball condition and many-standard control was considered as pure deviance detection, whereas the difference between the many-standard control and response to the final repetitive tone under the oddball condition was considered pure adaptation. In practice, an oddball paradigm consists of repeated pure-tone stimuli of the same frequency with a 1-s interval of stimulus onset, followed by a pure tone of the deviant frequency with a 10% chance. In turn, a many-standard control comprised 10 different frequencies of pure tones presented in a random order. Human MMN studies, which are generally conducted in awake individuals (*14*, *26*), have shown that MMN response is reduced under anesthesia (*27*). Therefore, we recorded the calcium responses evoked by auditory stimuli in awake mice.

The readout of the experiments needs to be reliable in the measurement of both population activity in specific subregions of the AC and single-neuron response. We first identified A1, AAF, and A2 using wide-field single-photon imaging (Fig. 2B) and compared the bulk calcium responses to auditory stimuli between control and Del(1.5 Mb)/+ mice (Fig. 2C). The average of trials in A1, AAF, and A2 in control mice indicated that the difference in calcium responses between the many-standard and oddball conditions was prominent in AAF and A2 (paired *t* test; AAF: *t* = 4.51, *P* = 0.0002; A2: *t* = 5.34, *P* < 0.0001) but was smaller and not significant in A1 (*t* = 0.56, *P* = 0.5820), suggesting the presence of a DD component in the higher-order AC (Fig. 2D). However, this component was no longer significant in AAF and A2 of Del(1.5 Mb)/+ mice (AAF: *t* = 2.02, *P* = 0.0616; A2: *t* = 1.33, *P* = 0.2019; A1: *t* = −0.79, *P* = 0.4424). Direct comparison of the deviance detection size between the genotypes revealed a significant reduction specifically in A2 of Del(1.5 Mb)/+ mice (*t* = 2.057, *P* = 0.0474) but not in A1 (*t* = 0.931, *P* = 0.3584) or AAF (*t* = 1.248, *P* = 0.2206).

We next evaluated the DD component in Del(1.5 Mb)/+ mice using two-photon calcium imaging at a temporal resolution of 30 Hz (a field of view of 508 μm by 508 μm, at a depth of 120 to 250 μm for L2/3 and 300 to 440 μm for L4/5). Our analysis focused on neurons that responded robustly to auditory stimuli, with their average responses of >3.5 SDs above baseline. We recorded 2257 tone-responsive neurons located in specific auditory subregions. By combining two-photon imaging of single-neuron activity with the paradigm of auditory stimulation described above, we successfully identified 401 (control mice) and 377 [Del(1.5 Mb)/+ mice] tone-responsive neurons in A1, 298 (control mice) and 434 [Del(1.5 Mb)/+ mice] tone-responsive neurons in AAF, and 395 (control mice) and 352 [Del(1.5 Mb)/+ mice] tone-responsive neurons in A2 [*n* = 6 mice for both WT and Del(1.5 Mb)/+]. Specifically, the mean response rate of individual mice was 6.44% [standard error of measurement (SEM), 1.87%] in the WT group and 6.54% (SEM, 1.50%) in the Del(1.5 Mb)/+ group. When further analyzed by auditory subfields, the response rates for WT and Del(1.5 Mb)/+ mice were 7.43% (SEM, 2.24%) and 5.52% (SEM, 1.55%) in A1, 6.67% (SEM, 3.50%) and 7.46% (SEM, 2.58%) in the AAF, and 6.69% (SEM, 1.44%) and 6.26% (SEM, 1.81%) in A2, respectively. In addition, the overall mean density of tone-responsive neurons was 27.05 cells/mm^2^ (SEM, 6.18 cells/mm^2^) in WT mice and 25.77 cells/mm^2^ (SEM, 5.62 cells/mm^2^) in Del(1.5 Mb)/+ mice. Across the specific subfields, the densities in WT and Del(1.5 Mb)/+ mice were 28.74 cells/mm^2^ (SEM, 9.59 cells/mm^2^) and 25.17 cells/mm^2^ (SEM, 6.56 cells/mm^2^) in A1, 30.03 cells/mm^2^ (SEM, 12.24 cells/mm^2^) and 29.32 cells/mm^2^ (SEM, 8.30 cells/mm^2^) in the AAF, and 26.13 cells/mm^2^ (SEM, 4.77 cells/mm^2^) and 20.84 cells/mm^2^ (SEM, 7.64 cells/mm^2^) in A2, respectively. The comparable density of tone-responsive neurons between control and Del(1.5 Mb)/+ mice further confirmed the unaltered ability of sound detection in the AC of Del(1.5 Mb)/+ mice.

The temporal pattern of calcium activity in single putative pyramidal neurons was recorded under both many-standard and oddball conditions (fig. S1A), and the population average of the calcium response showed a pattern similar to that of wide-field imaging (Fig. 2E). The presence of deviance detection (oddball minus control stimulus) was assessed through a comprehensive analysis of calcium responses in L2/3 and L4/5 across the three subareas (A1, AAF, and A2). In WT mice, both L2/3 and L4/5 neurons in AAF/A2 showed significant deviance detection (*t* test; L2/3: *t* = 13.06, *P* < 0.001; L4/5: *t* = 10.13, *P* < 0.001). However, in A1, only L2/3 neurons exhibited deviance detection (L2/3: *t* = 5.23, *P* < 0.001; L4/5: *t* = 1.87, *P* = 0.253), indicating weaker overall responses of A1 neurons to deviant signals (Fig. 2F). This large DD component in AAF/A2 L2/3 was significantly reduced in Del(1.5 Mb)/+ mice (Fig. 2G). Statistical significance for the regional and layer-specific impairment in AAF/A2 L2/3 was confirmed by a two-way analysis of variance (ANOVA) (genotype × region/layer interaction) [*F*(1, 2253) = 4.43, *P* = 0.035], followed by post hoc analysis showing a significant reduction in AAF/A2 L2/3 but not in other subareas. The basal responses of the many-standard controls were also evaluated (fig. S1B). No significant differences were observed between control and Del(1.5 Mb)/+ mice, indicating a specific alteration in the DD component in Del(1.5 Mb)/+ mice.

The reduced amount of DD component in Del(1.5 Mb)/+ mice could result from impaired thalamic inputs to AAF/A2. To test this possibility, we performed in vivo two-photon imaging of thalamocortical axonal boutons in the AC (fig. S1C). In both L1 and L3/4, the boutons of thalamic axons showed larger differences in calcium responses between the many-standard and oddball conditions in AAF/A2 than in A1, similar to the tendency of somatic calcium responses (Fig. 2, H and I, and fig. S1D). Specifically in WT mice, oddball responses were significantly enhanced in both L1 (*t* = 11.50, *P* < 0.001) and L3/4 (*t* = 10.20, *P* < 0.001) of AAF/A2, whereas in A1, significant enhancement was limited to L1 (*t* = 3.53, *P* = 0.002; L3/4: *t* = 2.12, *P* = 0.148). This large component of the deviance detection in AAF/A2 was preserved in Del(1.5 Mb)/+ mice. These results are consistent with the idea that thalamic inputs to AAF/A2 are not the primary cause of impaired deviance detection in the postsynaptic neurons of AAF/A2 in Del(1.5 Mb)/+ mice.

### Reduced clustering of DD neurons in AAF/A2 of Del(1.5 Mb)/+ mice

**Fig. 3.**
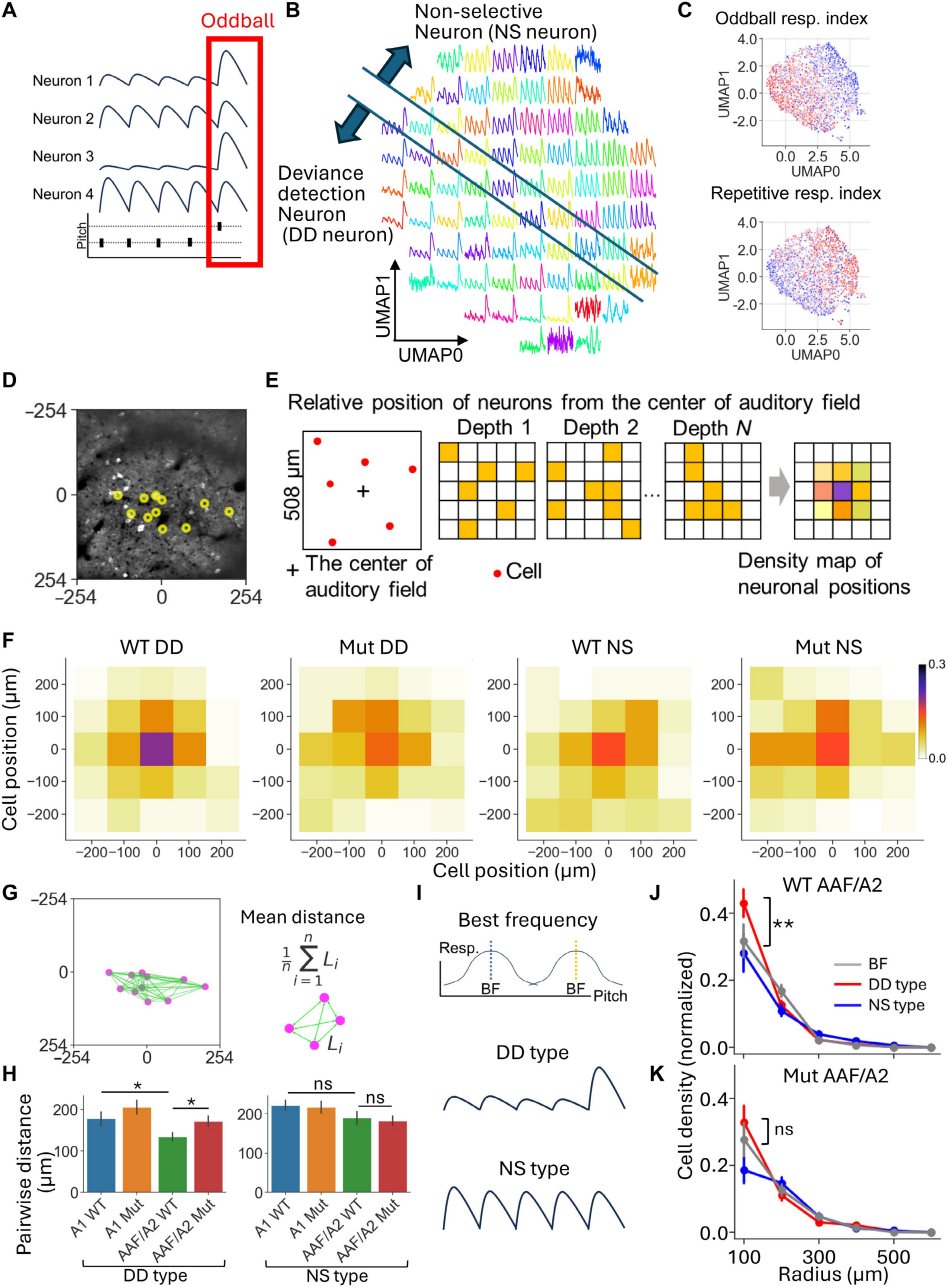
Reduced clustering of DD neurons in AAF/A2 of Del(1.5 Mb)/+ mice. (**A**) Representative calcium response to four repetitive and the following deviant tones. (**B**) Calcium response patterns in a 10 by 10 grid within UMAP. DD and NS neurons were below or above the two oblique lines. (**C**) Oddball and repetitive response indices, color-coded from blue (low) to red (high), within UMAP. (**D**) Spatial distribution of DD neurons (yellow circles) overlaid on the time-averaged fluorescence for structural landmarks. Axis unit: micrometers. (**E** and **F**) Spatial clustering evaluated by cell density maps. (E) Cell positions relative to the auditory subfield centers integrated across multiple depths. (F) Color-coded cell density map of L2/3 neurons in AAF/A2. Rostro-caudal and ventral-dorsal directions are along horizontal and vertical axes, respectively. (**G** and **H**) (G) Calculation of pairwise distance (green lines) between neurons (pink dots). Axis units: micrometers. (H) Shorter pairwise distance of WT DD neurons in AAF/A2 than in A1 (left); significant difference among groups [*F*(3, 122) = 4.45, *P* = 0.0053], post hoc Bonferroni-corrected *t* test between A1 and AAF/A2 (*P* = 0.041). This difference between AAF/A2 and A1 is not observed in Mut AC. NS neurons show no significant differences (right) [one-way ANOVA, *F*(3, 117) = 1.69, *P* = 0.174]. (**I** to **K**) Spatial distribution of DD, NS, and frequency-responsive neurons in AAF/A2. (I) Best frequency–based grouping into 10 groups (BF neurons), followed by calculation of mutual distances, in comparison with mutual distances of DD and NS neurons. [(J) and (K)] Cell fractions against mutual distance indicate the trend of higher cell fractions with distances of <200 μm in all neuron groups. Note significant difference between BF and DD neurons with distances of <100 μm, confirmed by a Mann-Whitney test. *P < 0.05 and **P < 0.01. For the sample numbers, see Supplementary Data for Statistical Analysis.

To visualize the variation in the response waveforms of neurons to the four repetitive stimuli and subsequent deviant stimuli in an unbiased manner, we applied Uniform Manifold Approximation and Projection (UMAP) (*28*) to the calcium response curves from 2946 neurons in both L2/3 and L4/5 (1734 neurons for L2/3 and 1212 neurons for L4/5), all three subfields (993 neurons for A1, 926 neurons for AAF, and 1027 neurons for A2), and both genotypes [1428 neurons for WT mice and 1518 neurons for Del(1.5 Mb)/+ mice] (Fig. 3, A and B, and fig. S2A). We found that the response patterns of neurons were distributed without clear boundaries. However, a steady shift in the calcium response pattern was detected in an oblique direction from the bottom left to top right. To further clarify this point, we divided the projected data space into 10 × 10 subareas and plotted the representative calcium response in each subarea (Fig. 3B). The overall distribution of the calcium response pattern confirmed the gradual transition from neurons specifically responding to the last oddball tone (bottom left subareas) to neurons equally responding to all five tones (top right subareas; Fig. 3C). We took advantage of this directional transition and classified the total neuronal responses into three groups: DD neurons, nonselective (NS) neurons, and remaining intermediate neurons, with their relative positions along the oblique direction in the UMAP projection plane (Fig. 3B). The fractions of DD neurons within the tone-responsive neurons were 31.4% (control mice) and 21.4% [Del(1.5 Mb)/+ mice] in A1 and 52.7% (control mice) and 43.6% [Del(1.5 Mb)/+ mice] in AAF/A2, indicating a lower number of DD neurons in Del(1.5 Mb)/+ mice than in control mice.

Next, we analyzed whether there was any difference in the distribution patterns of DD and NS neurons in L2/3 of AAF/A2. For this purpose, we generated a cell density map, with the cell positions measured relative to the center of the auditory field (Fig. 3, D and E). In control mice, DD neurons were distributed at a high density around the center (Fig. 3F). This clustering tendency was less evident in the density map of NS neurons. The density of DD neurons around the center was highly reduced in Del(1.5 Mb)/+ mice, and the difference between DD and NS neurons was no longer detectable. To quantify the tendency of neuron clustering, we first calculated the fraction of neurons present within 100 μm of the center of the auditory subfields. The calculated fraction in WT mice was higher for DD neurons (47.5%) than for NS neurons (34.9%) in AAF/A2. However, this difference was not evident in Del(1.5 Mb)/+ mice (DD neurons, 33.8%; NS neurons, 33.5%). The cumulative frequency plots of the distance between neurons and the center of AAF/A2 also confirmed the difference between WT and Del(1.5 Mb)/+ mice (fig. S2B). As another indicator of spatial clustering, the distances between all possible pairs of neurons were calculated (Fig. 3G). The mean distance between DD neurons in AAF/A2 of the control mice was smaller than that in A1 (Fig. 3H). Again, this difference was not evident in Del(1.5 Mb)/+ mice, supporting the notion that the spatial distribution of DD neurons in AAF/A2 is disrupted in Del(1.5 Mb)/+ mice.

If the frequency tunings of DD neurons are similar and neurons with similar best frequencies tend to cluster, then the high density of DD neurons around the center can be explained solely by their frequency tuning. To rule out this possibility, we studied the extent of clustering in neurons with similar best frequencies by measuring the pairwise distances of neurons within the same frequency band. Specifically, we categorized neurons into 10 subgroups based on their best frequencies and calculated the mutual distances between neurons within each subgroup (Fig. 3I). The fraction of neuron pairs with similar best frequencies decreased gradually as the distance increased (Fig. 3J). The fraction of DD neuron pairs located within 100 μm was significantly higher than that of neurons with similar best frequencies in AAF/A2. This analysis indicated the existence of an independent clustering mechanism for DD neurons in the AAF/A2. Consistent with the analysis of the cell density map in Fig. 3F, DD neuron clustering in Del(1.5 Mb)/+ mice was attenuated to the level of neurons with a similar best frequency (Fig. 3K). Furthermore, statistical comparison of the cell fraction within a 100-μm radius revealed a DD neuron–specific reduction in Del(1.5 Mb)/+ mice (*P* = 0.0323), whereas NS neurons showed no significant difference (*P* = 0.2992). This indicates that DD neurons are selectively susceptible to the mutation within local networks, which is consistent with the overall pairwise distance analysis (Fig. 3H). In A1, the fraction of DD neuron pairs located within 100 μm was comparable to that of neurons with similar best frequencies. Furthermore, no difference in A1 was observed between control and Del(1.5 Mb)/+ mice. These results indicate that DD neuron clustering in AAF/A2 is driven by a mechanism distinct from the tonotopic patterning of AC.

### DD neurons show higher local functional connectivity in AAF/A2

**Fig. 4.**
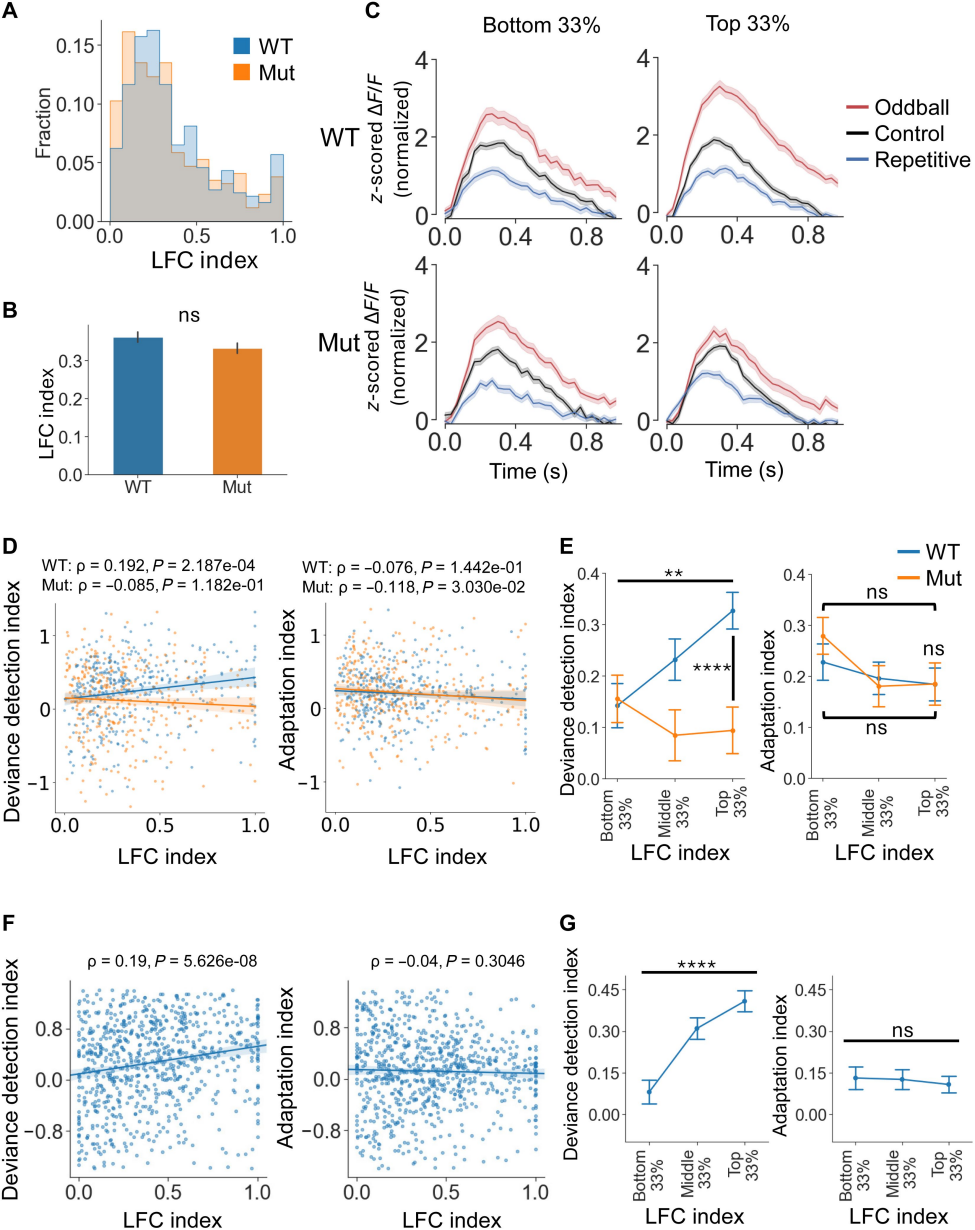
DD neurons show high local functional connectivity in AAF/A2. (**A** and **B**) Similar distributions (A) and mean (B) of the LFC index of neurons in WT (blue) and Mut (orange) mice. (**C**) Larger deviance detection signals in neurons with higher LFC indices in the AAF/A2 and their attenuation in Mut neurons. The population-averaged calcium responses of single neurons to oddball stimuli (red), control stimuli (black), and repetitive stimuli (blue) were normalized to the responses to control stimuli (solid lines, mean responses; shaded areas, SEM). Neurons were grouped according to the LFC index into either the bottom or top 33% of the groups. (**D**) Relationship between the LFC index and deviance detection index (left) and adaptation index (right). Each dot represents a single neuron. Colors indicate genotypes (WT, blue; Mut, orange). Linear regression lines with a 95% confidence interval (shaded areas) are shown with Spearman’s ρ and *P* values. (**E**) Higher deviance detection index in WT neurons in the top 33% of the LFC index (left). This relationship is disrupted in Mut neurons. The adaptation and LFC indices do not correlate in either WT or Mut neurons (right). (**F**) Relationship between the LFC index and either the deviance detection index (left) or adaptation index (right) using the LFC index calculated from calcium activity without tone presentation in WT mice. Linear regression lines with a 95% confidence interval (shaded areas) are shown with Spearman’s ρ and *P* values. (**G**) Higher LFC indices calculated from calcium activity without tone presentation are effective in grouping neurons with a high deviance detection index (left), whereas the adaptation index does not show a correlation with the LFC index without stimuli (right). For the sample numbers, see Supplementary Data for Statistical Analysis. Statistical evaluation was performed using Student’s *t* test for (B), (E), and (G). **P < 0.01 and ****P < 0.0001.

Analysis of the spatial distribution of tone-responsive neurons in AAF/A2 revealed preferential clustering of DD neurons. The proximity of the DD neuron population may cause correlated firing via higher synaptic connectivity within local cortical circuits. To identify local functional ensembles, we tested for the presence of correlated firing between neuron pairs during the period of tone presentation. We calculated the index of local functional connections (LFCs) for all imaged neurons. The LFC index was calculated from the correlation coefficient of the calcium activity between the target neuron and nearby neurons positioned within a distance of 50 μm. This threshold was selected to conservatively capture immediate neighbors within locally connected microcircuits with high synaptic connection probabilities (*29*). The obtained correlation coefficients were normalized, and the top 10% values were averaged to obtain the LFC index for the target neuron. The distribution of the LFC index in the AAF/A2 showed a skewed distribution with a peak around 0.25, indicating that neurons with high functional connectivity were infrequent. The overall distribution and mean of the LFC index were comparable between control and Del(1.5 Mb)/+ mice (Fig. 4, A and B).

Neurons with high and low LFC indices may show different responses to tone presentation under both the adaptation and oddball conditions. The averaged responses of single neurons that belonged to the bottom and top one-third of the LFC index showed distinct patterns, with a prominent oddball response in neurons with a high LFC index and moderate response in neurons with a low LFC index (Fig. 4C). However, the strong oddball response in neurons with a high LFC index was completely abolished in Del(1.5 Mb)/+ mice. The response to tone under the adaptation condition was similar for neurons with different LFC indices and genotypes. To further reveal the relationship between local functional connectivity and response under oddball conditions, we plotted the extent of deviance detection against the LFC index. There was a positive correlation in control mice; however, this relationship was not observed in Del(1.5 Mb)/+ mice (Fig. 4D, left). In contrast, the extent of adaptation was not positively correlated with the LFC index in either genotype (Fig. 4D, right). To further clarify the population differences in the relationship between the LFC index and size of deviance detection, we calculated the average deviance detection in neuronal populations grouped according to the value of the LFC index. Neurons with high LFC indices responded strongly to oddball tones (with a high deviance detection index) (Fig. 4E, left). This correlation indicates that neurons that can detect deviant signals utilize strong functional connectivity with nearby neurons for the recurrent amplification of excitation. This enhanced response to oddball tones was eliminated in Del(1.5 Mb)/+ mice, further supporting the close relationship between deviance detection and LFCs. A similar plot for the extent of adaptation against the LFC index did not reveal a difference between the control and Del(1.5 Mb)/+ mice, indicating intact adaptation mechanisms in Del(1.5 Mb)/+ mice (Fig. 4E, right).

So far, the LFC index was calculated from calcium transients during the period of tone presentation. Temporally correlated responses may be derived either from responses to stimuli or from intrinsic spontaneous activity. To test whether neurons judged to be locally connected from intrinsic spontaneous activity also show a strong oddball response, we repeated the analysis using calcium activity during the period without tone presentation. The overall relationship between the LFC index and extent of deviance detection, together with the high deviance detection index in the neuron group with a high LFC index, was reproduced from the calcium activity under the resting condition (Fig. 4, F and G). This result suggests that intrinsically synchronized network scaffolds may promote the detection of deviant signals.

### Molecular profiling of L2/3 neurons in AAF/A2 of Del(1.5 Mb)/+ mice

**Fig. 5.**
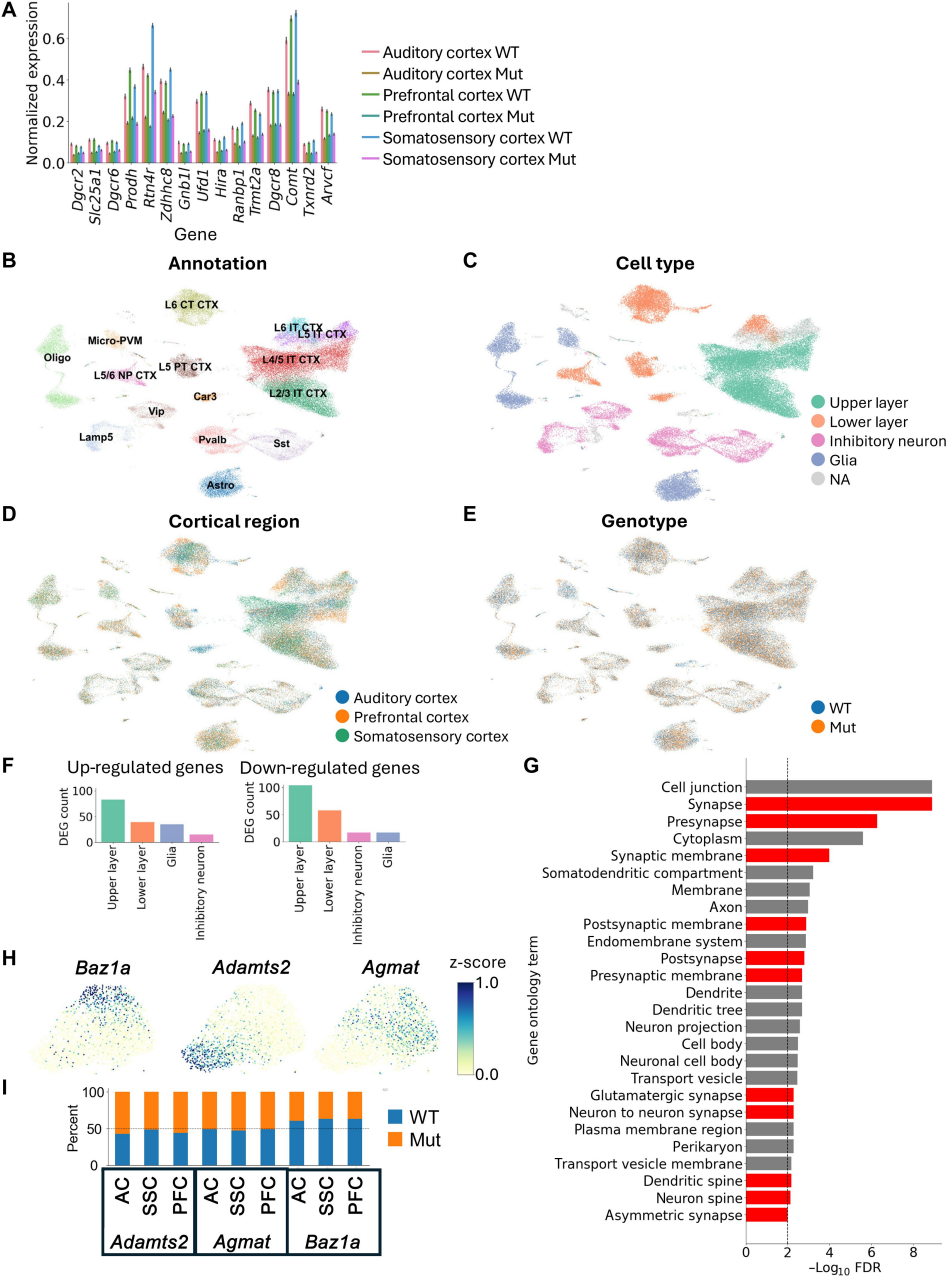
Molecular profiling of L2/3 neurons in AAF/A2 of Del(1.5 Mb)/+ mice. (**A**) Reduced expression of gene subsets located in deletion regions (1.5 Mb) that are heterozygous in Mut mice. (**B** to **E**) UMAP representation of single-nucleus transcriptomic data showing cell clusters with annotations (B), cell types (C), cortical regions (D), and genotypes (E). (**F**) Number of DEGs identified in each cell type. DEGs are defined as genes with a log_2_ fold change of >0.3 (up-regulated) or <−0.3 (down-regulated) and an FDR of <0.01. (**G**) GO term analysis of DEGs in the upper-layer excitatory neurons of WT and Mut mice. GO terms are ranked according to the FDR, with the dashed line indicating FDR = 0.01. The red bars indicate the GO terms related to synapses. (**H**) Mutually exclusive patterns of expression of the three markers (*Baz1a*, left; *Adamts2*, middle; *Agmat*, right) in the population of L2/3 IT CTX neurons in the UMAP plot. Gene expression levels are expressed as *z*-scores. (**I**) Proportions of WT and Mut neurons for each neuron subtype and region after normalizing the number of cells across the batches. A chi-square test was performed for each brain region using the counts of *Baz1a*-positive and *Baz1a*-negative cells across genotypes, and the differences were found to be statistically significant (AC: χ^2^ = 33.47, *P* < 0.0001, PFC: χ^2^ = 65.59, *P* < 0.0001; SSC; χ^2^ = 42.33, *P* < 0.0001). For the sample numbers, see Supplementary Data for Statistical Analysis.

Our analysis of neuronal responses to oddball stimuli in the AC provides evidence for the distinct local circuit property in L2/3 of AAF/A2 and its dysfunction in Del(1.5 Mb)/+ mice. Altered circuit properties in the mouse model could be derived from altered gene expression in specific cell types. To reveal the differences in gene expression profiles in the AC of control and Del(1.5 Mb)/+ mice, we performed single-nucleus RNA sequencing (snRNA-seq) to examine the transcriptional profiles of individual cells from the AC, prefrontal cortex (PFC), and somatosensory cortex (SSC) (*30*–*35*). We sequenced 30,741 cells in the control AC, 15,831 cells in the control PFC, 13,359 cells in the control SSC, 11,779 cells in the Del(1.5 Mb)/+ AC, 13,802 cells in the Del(1.5 Mb)/+ PFC, and 12,693 cells in the Del(1.5 Mb)/+ SSC (four male animals at the age of 19 weeks postnatal for each genotype). We removed low-quality sequence data, possibly derived from debris, doublets/multiplets, and dead cells. After the quality control step, we identified 8506 cells in the control AC, 12,049 cells in the control PFC, 10,293 cells in the control SSC, 9113 cells in the Del(1.5 Mb)/+ AC, 10,677 cells in the Del(1.5 Mb)/+ PFC, and 9774 cells in the Del(1.5 Mb)/+ SSC. Consistent with the deletion of the chromosomal region corresponding to human 22q11.2, the expression levels of genes within the deleted region were invariably reduced (Fig. 5A). Unsupervised clustering analysis of the combined sequence data identified 29 cell types with distinct expression profiles, of which 15 had a cell count that represented ≥1% of the total population (Fig. 5B). We focused on the differential gene expression in the upper-layer excitatory neurons, corresponding to the cell types of L2/3 intratelencephalic cortex (IT CTX) and L4/5 IT CTX (Fig. 5C). The excitatory neurons in the upper layer showed mild differences in gene expression profiles among the three cortical areas (Fig. 5D). Control and Del(1.5 Mb)/+ mice did not show a marked difference in the general pattern of the cell types (Fig. 5E). Although the number of excitatory cells in the cortical upper layer was comparable between the genotypes (figs. S3A and S4B), the identified differentially expressed genes (DEGs) were enriched in the upper-layer excitatory neurons (Fig. 5F and fig. S3B). Gene ontology (GO) analysis of the upper-layer excitatory neurons revealed gene enrichment related to synaptic structure and function, such as synapse [false discovery rate (FDR) = 1.254 × 10^−9^], presynapse (FDR = 5.282 × 10^−7^), and postsynaptic membrane (FDR = 1.289 × 10^−3^) (Fig. 5G), strongly supporting the altered synaptic functions in this neuron population of Del(1.5 Mb)/+ mice.

In the mouse visual cortex, two subclasses of L2/3 neurons, positive for the marker genes *Adamts2* and *Baz1a*, were shown to be activated by stimuli that elicit negative and positive prediction error responses, respectively (*36*). A similar relationship may exist between L2/3 neuron subclasses in AC and their responses to auditory oddball conditions. Therefore, we classified the upper-layer excitatory neurons into three transcriptionally defined subclasses (Fig. 5H) and calculated the relative abundance between control and Del(1.5 Mb)/+ mice for each subclass (Fig. 5I). In L2/3 IT CTX cells, the proportion of the *Baz1a* subclass was reduced in Del(1.5 Mb)/+ mice (13.83%, 739/5342 cells) compared to WT mice (22.68%, 1137/5014 cells). Conversely, the proportions of the *Adamts2* and *Agmat* subclasses were relatively preserved or slightly increased in the mutant mice (*Adamts2*: 32.12%, 1716/5342 cells versus 26.88%, 1348/5014 cells; *Agmat*: 54.04%, 2887/5342 cells versus 50.44%, 2529/5014 cells). It has been proposed that neurons responding to positive prediction error responses receive direct sensory input to drive their activities (*37*). This model fits our observation of reduced oddball responses in Del(1.5 Mb)/+ mice with reduced *Baz1a* neurons, which serve to detect the presence of sensory input without predictions.

To reveal the distribution of different cell types and gene expression profiles, we performed spatial transcriptomic analysis using the Xenium In Situ Platform (10x Genomics) in L2/3 of the WT and the Del(1.5 Mb)/+ AC (fig. S4, A to C). The total cell counts were 125,565 for the WT and 127,840 for the Del(1.5 Mb)/+. Within the L2/3 regions, the total cell counts were 2143 (area: 724,519.68 μm^2^) in WT and 2127 (area: 730,345.45 μm^2^) in Del(1.5 Mb)/+. The density of the *Baz1a* subclass in WT mice (*n* = 356) was 491.36 cells/mm^2^, whereas it was reduced to 321.77 cells/mm^2^ in Del(1.5 Mb)/+ mice (*n* = 235; 0.65-fold relative to WT), consistent with the decrease in cellular proportion observed in the snRNA-seq data (fig. S4D). In contrast, no clear differences in cell density were observed for the *Adamts2* or *Agmat* subclasses (*Adamts2*: WT, *n* = 457, 630.76 cells/mm^2^; Del(1.5 Mb)/+, *n* = 432, 591.50 cells/mm^2^; *Agmat*: WT, *n* = 581, 801.91 cells/mm^2^; Del(1.5 Mb)/+, *n* = 692, 947.50 cells/mm^2^). To confirm the reduced density of the *Baz1a* subclass, we next calculated the nearest-neighbor distance (NND). The mean NND of *Baz1a* subclass cells was 26.39 μm in WT mice but was increased to 32.79 μm in Del(1.5 Mb)/+ mice (1.24-fold relative to WT) (fig. S4, E and F). No large changes in NND were detected for the other subclasses (*Adamts2*: WT, 20.65 μm; Del(1.5 Mb)/+, 21.26 μm; *Agmat*: WT, 20.72 μm; Del(1.5 Mb)/+, 20.05 μm). In addition, IEG expression across subtypes revealed that, regardless of genotype, *Baz1a* subclass exhibited a higher coexpression rate of IEGs than the other subclasses. For example, in WT mice, the coexpression rate of *Fos* was 9.3% in the *Baz1a* subclass, compared with 0.22% in the *Adamts2* subclass and 0.69% in the *Agmat* subclass. A similar trend was observed for other IEGs, including *Arc*, *Fosl2*, *Npas4*, and *Nr4a2* (fig. S4G). These results indicate that the *Baz1a*-positive neurons, which express high IEGs, reduce their density and spatial clustering in Del(1.5 Mb)/+ mice.

### Spine synapse phenotypes in c-fos–positive L2/3 pyramidal neurons

**Fig. 6.**
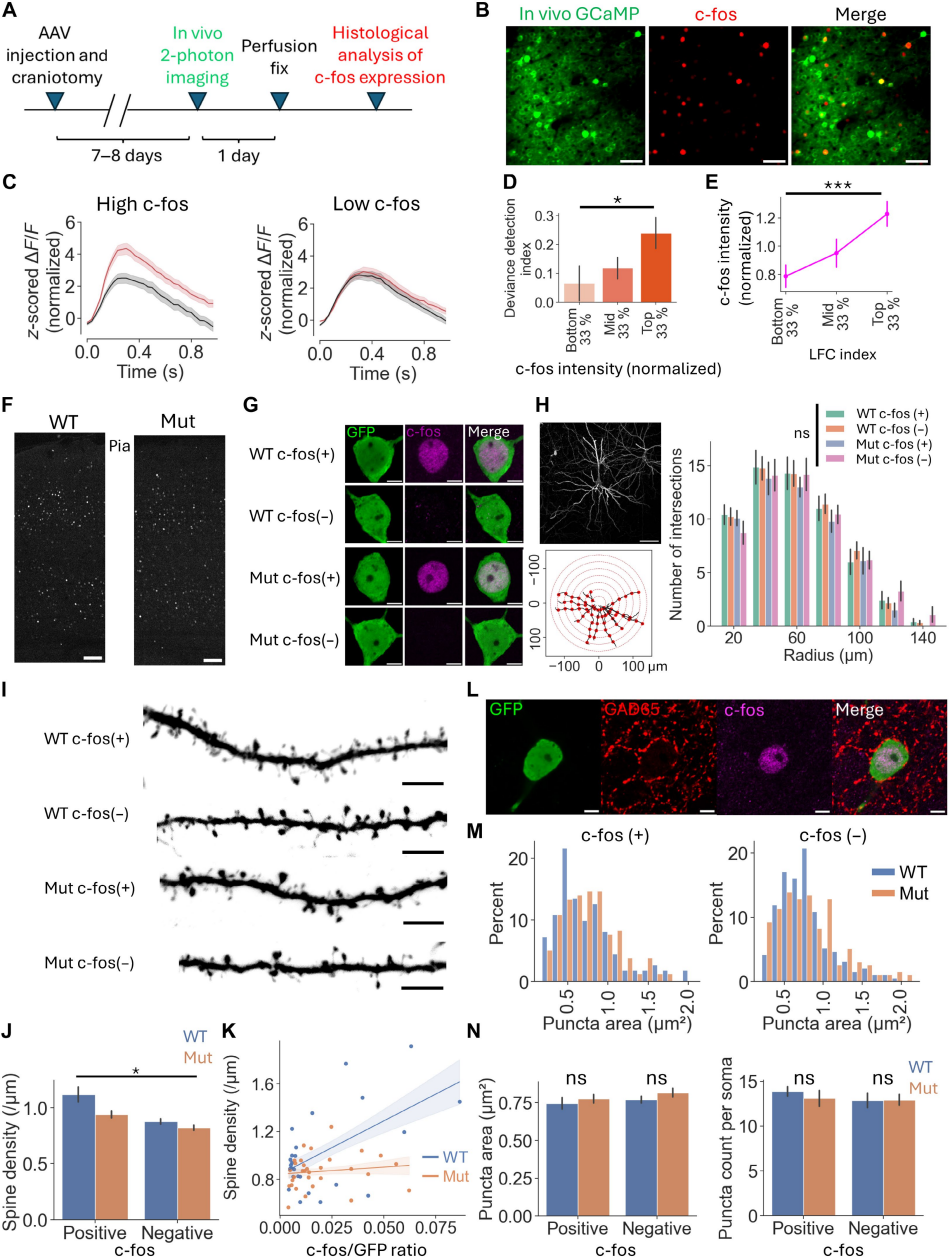
Spine synapse phenotypes in c-fos–positive L2/3 pyramidal neurons. (**A** to **E**) Calcium imaging and post hoc identification of c-fos–positive neurons in AAF/A2 of WT mice. (A) Time-course of experimental procedures. (B) In vivo calcium imaging with post hoc anti–c-fos immunostaining. (C) Responses to oddball (red) and control (black) stimuli for neuron groups with the top (“high c-fos”) and bottom 33% (“low c-fos”) c-fos intensity. (D) Strong deviance detection in neurons with top 33% c-fos intensity. (E) Higher c-fos staining in neurons with top 33% LFC index. (**F**) A similar distribution of c-fos (+) neurons in WT (left) and Mut (right) AC. The pial surface labeled as “pia.” (**G**) Images of c-fos (+) and (−) cell bodies manually identified based on immunoreactivity. (**H**) Maximum projection image of a c-fos (+) WT pyramidal neuron in AAF/A2 (top left) and dendrite tracing (bottom left), with intersection points of dendrites and circles of different radii (red dots). The number of intersection points plotted against distance (right), with no significant difference (two-way ANOVA). (**I** to **K**) Spine density analysis. (I) Images of dendritic segments with spines. (J) Spine density of different neuron groups revealed a significant interaction between c-fos and genotype (*P* = 0.036), along with a main effect of c-fos (*P* = 0.012). (K) Plot of spine density and c-fos/GFP intensity ratio indicates WT-specific interaction between genotype and the c-fos/GFP ratio (*P* = 0.021). (**L** to **N**) GAD65 (red) and -c-fos (magenta) immunostaining of GFP-positive neurons (green) identified cell body–associated inhibitory boutons (L), with similar size distributions (M), puncta sizes and numbers (N), between groups. Student’s *t* test [(D), (E), and (N)] and a linear mixed-effects model [(J) and (K)]. Shaded areas in (C) and ( K) indicate SEM. Scale bars, 50 μm [(B) and (H)], 100 μm (F), and 5 μm [(G), (I), and (L)]. *P < 0.05 and ***P < 0.001. For the sample numbers, see Supplementary Data for Statistical Analysis.

A previous study of mouse SSC reported that *Baz1a*-positive neurons in the upper cortical layer effectively detected tactile features and showed enrichment of plasticity-related IEGs (*38*). Consistent with this finding, the expression patterns of several IEGs also showed enrichment of *Baz1a*-positive neurons among the upper-layer excitatory neurons in the AC (figs. S3, C and D, and S4G). To test the relationship between c-fos (representative IEG) expression and neuronal responses in the oddball condition, we performed post hoc immunohistochemistry of c-fos in cortical samples taken from mice previously subjected to in vivo two-photon calcium imaging to detect DD neurons (Fig. 6, A and B). Calcium responses under the control and oddball conditions for c-fos (+) and c-fos (−) neurons were markedly different (Fig. 6C). The population average of the extent of deviance detection between groups categorized according to c-fos intensity indicated a high ability to detect deviant signals in neurons with strong c-fos expression (Fig. 6D). This correlation was not due to c-fos induction by auditory stimulation, as the sampling of AC was performed the day after in vivo calcium imaging when the effect of c-fos induction by auditory stimulation was negligible (*39*, *40*). Moreover, c-fos expression level was positively correlated with the LFC index (Fig. 6E), suggesting that neurons with sustained c-fos expression had a strong connectivity with the surrounding neurons.

To further delineate the cellular properties of c-fos–positive neurons, we characterized dendritic arborization, spine synapse density, and inhibitory synapse density in both control and Del(1.5 Mb)/+ mice, using sparse labeling with a mixture of AAV-Cre and AAV–flip-excision (FLEX)–green fluorescent protein (GFP), followed by immunostaining. The overall pattern and density of c-fos (+) cells in the AC were comparable between genotypes (Fig. 6, F and G). Dendritic complexity, evaluated using the Sholl analysis, also did not show any noticeable differences (Fig. 6H). However, the density of excitatory spine synapses was significantly reduced in c-fos (+) neurons of Del(1.5 Mb)/+ mice (Fig. 6, I and J). Spine density positively correlated with the relative intensity of c-fos immunoreactivity in control mice; however, this correlation was not observed in Del(1.5 Mb)/+ mice (Fig. 6K). The density and size of inhibitory synapses, measured using immunostaining with anti-GAD65 (a marker of inhibitory presynaptic boutons), were not altered in Del(1.5 Mb)/+ mice, irrespective of the presence of c-fos signals (Fig. 6, L to N). Together, these results indicate that c-fos (+) upper-layer neurons in Del(1.5 Mb)/+ mice are specifically impaired in excitatory synaptic transmission, which may cause reduced activity in response to the oddball stimulus.

### Negative top-down feedback from higher AC to lower AC was not observed in the 22q11.2 deletion syndrome mouse model

**Fig. 7.**
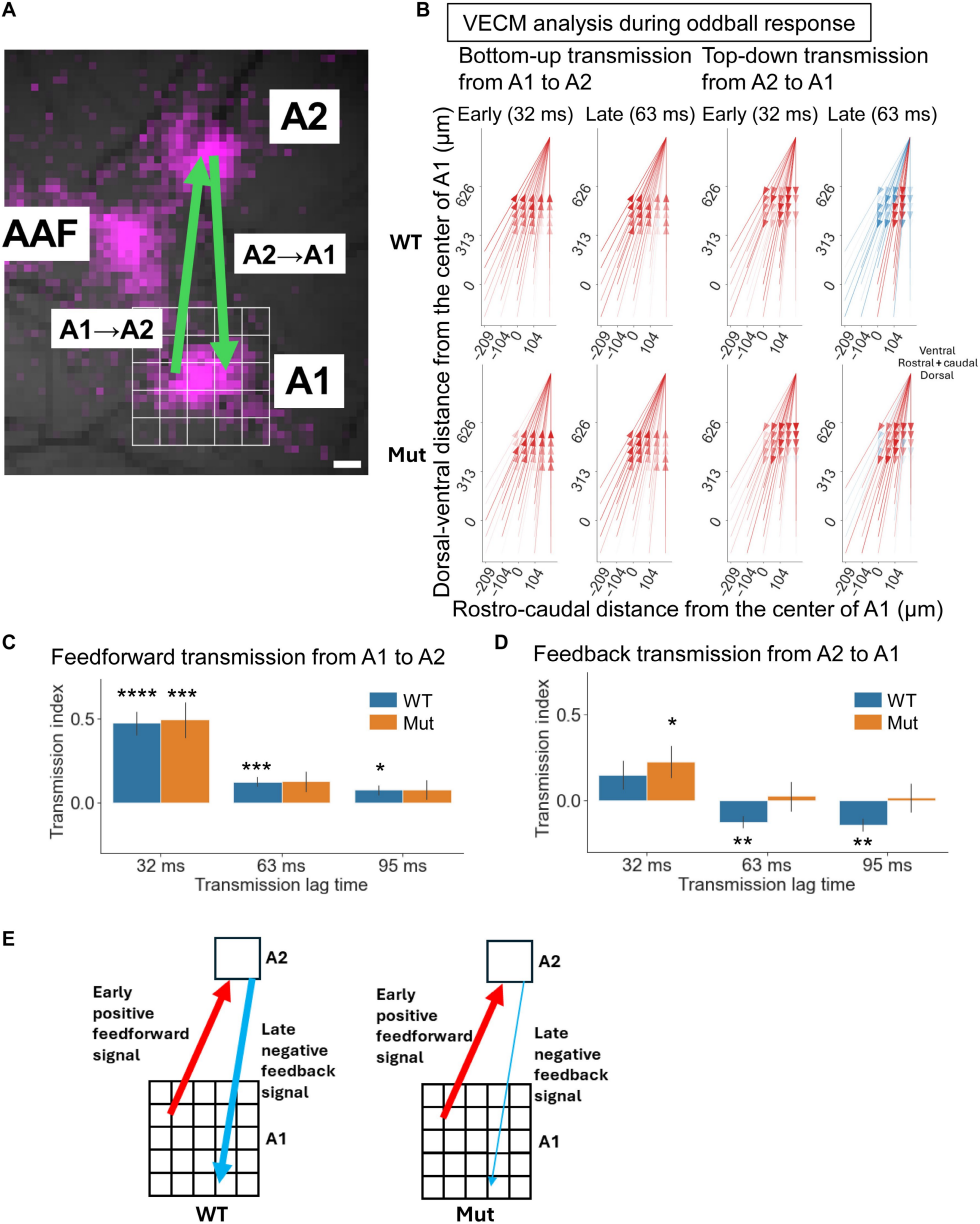
Negative top-down feedback from higher AC to lower AC and its impairment in Del(1.5 Mb)/+ mice. (**A**) Locations of A1, AAF, and A2 in the image obtained by wide-field imaging of the right AC expressing GCaMP8m. Signal propagation between A1 and A2 during auditory stimulation in the MMN paradigm was analyzed. The 5 by 5 subregions were set in A1 for their interactions with A2. (**B**) Population average of the feedforward signal from A1 to A2 (left) and feedback signal from A2 to A1 (right) during the oddball response in WT (top) and Mut (bottom) mice, with signal transmission latencies of 32 and 63 ms. The direction of the arrowheads on the 5 by 5 subregions of A1 indicates whether the signals are feedforward or feedback. The intensities of the arrowheads and lines indicate signal sizes. The colors of arrowheads and lines indicate positive (red) or negative (blue) effects on the target areas. Negative effects are observed in feedback signals during the late phase in WT mice. (**C** and **D**) Feedforward (C) and feedback (D) signals between A1 and A2, with transmission latencies of 32 ms (left), 63 ms (middle), and 95 ms (right) during oddball responses. (**E**) Model of bidirectional signal propagation. The early positive bottom-up feedforward signal from A1 to A2 is intact in Mut mice, whereas the late negative top-down feedback signal from A2 to A1 is impaired. For the sample numbers, see Supplementary Data for Statistical Analysis. Statistical evaluation was performed using Student’s *t* test in (C) and (D). **P < 0.01, ***P < 0.001, and ****P < 0.0001. Scale bar, 100 μm (A).

The deviance detection signal, which presents prediction errors and is generated in the L2/3 neuron network of AAF/A2, serves as a critical output for further feedforward or feedback interactions with other cortical areas. The feedforward spread of prediction errors from the AC to higher-order cortical regions has been proposed to have multiple functions, including the detection of complex structures in sensory information, integration of multiple sensory information with different modalities, and behavioral choice. However, the functional role of feedback prediction errors in AC has not been clarified. In the marmoset AC, the prediction error signal generated in the higher-order AC functions as a positive feedback signal for A1, enhancing the A1 response to the oddball signal (*9*). To reveal the functional role of the prediction error in AAF/A2 in the A1 response, we used wide-field one-photon imaging to simultaneously detect calcium signals from both the lower-order (A1) and higher-order (AAF and A2) ACs (Fig. 7A). This experimental setup successfully detected attenuated deviance detection signals in A2 of Del(1.5 Mb)/+ mice, as previously described (Fig. 2D).

We applied vector error correction models (VECMs) to explore the structure of signal transmissions between higher- and lower-order ACs (Fig. 7B) (*41*–*43*). VECM can be applied to a system containing multiple dynamic components that interact with each other (*44*). The interactions are nonstationary but move toward equilibrium. The recurrent interaction between A1 and AAF/A2 initiated by sound presentation can be effectively analyzed using VECM, which explains the temporal changes in the propagation of activity between the components. We constructed VECMs that describe the A1-A2 interaction or A1-AAF interaction using calcium response data from 5 by 5 grids in A1 against the entire activity of either A2 or AAF. The validity of the VECM was confirmed by performing a cointegration test. At a transmission latency of 32 ms (i.e., the first imaging frame), positive propagation from A1 to A2 was observed for oddball stimuli in both WT and Del(1.5 Mb)/+ mice (Fig. 7, B and C). At latencies of 63 and 95 ms (i.e., the second and third frames, respectively), negative propagation from A2 to A1 was observed in WT mice; however, no significant negative propagation was observed in Del(1.5 Mb)/+ mice for oddball stimuli (Fig. 7D). In contrast, negative feedback signals were not observed between A1 and AAF (fig. S5), emphasizing the essential and specific role of A2 in the higher-order AC. These results suggest the presence of delayed top-down negative-feedback deviance detection signals, which may suppress the generation of error signals in A1 (Fig. 7E). This negative regulation by A2 may help differentiate the output from A1 and A2, with A1 conveying pure-tone signals and AAF/A2 conveying error signals.

## DISCUSSION

In this study, we investigated the mechanism of reduced deviance detection in the AC of a mouse model of schizophrenia. We thoroughly investigated multiple AC subregions spanning from the primary to higher auditory fields and from the superficial to deep cortical layers. We found that deviance detection was significantly enhanced in L2/3 neurons of the higher AC. Neurons that detected deviant tones were spatially clustered, functionally connected strongly with nearby neurons, and showed a higher expression of IEGs. This specific neuron network present in the higher AC generates negative feedback to A1 and may support the differentiation of the signals generated in the lower- and higher-order AC into either tone-evoked signals or context-related error signals, respectively. In this mouse model of schizophrenia, the expression of synapse-related genes was dysregulated, the postsynaptic organization in L2/3 pyramidal neurons was impaired, and the spatial clustering and functional connection of DD neurons were reduced, leading to significant attenuation of error signals generated in higher-order areas of the AC. We postulate that similar network alterations are present in patients with schizophrenia, causing pathological changes in auditory information processing.

We provide an unbiased evaluation of the mismatch responses using two-photon calcium imaging of multiple areas of the AC. Previous studies on rodent AC have reported the presence of hierarchical features in the detection of deviant tones, with a greater extent of deviance detection in higher-order areas of AC than in lower-order areas (*10*, *45*). Consistent with these findings, our imaging analysis revealed the prominent deviance detection in L2/3 of AAF/A2. We took advantage of recording a large number of excitatory neurons in individual subregions, leading to the identification of spatially clustered DD neurons with high correlation in activity. In our analysis, we categorized one-third of the tone-responding neurons as DD neurons. Although the transition of responses between DD neurons and others is gradual, the threshold for this categorization is in agreement with the results of the manual classification. Consistently, a previous study on DD neuron identification in V1 also reported that approximately one-third of visually driven neurons were deviance detectors (*46*). In the marmoset rostral parabelt, a higher-order AC generating pure error signals, the proportion of neurons that responded to deviant tones was 65%, which was higher than the ratios in the mouse sensory cortices (*9*). This difference may be related to the specialized function of the marmoset rostral parabelt in generating the error signals.

Two-photon in vivo imaging of neurons in the sensory cortex is a powerful approach for elucidating the computational role of single neurons in deviance detection (*47*). In the paradigm of sensory-motor mismatch, dissociation between self-generated movements and sensory inputs was experimentally induced, and the neurons that responded to the mismatch were analyzed (*48*). This approach identified two types of neurons that respond to sensory-motor mismatch, positive prediction error neurons and negative prediction error neurons (*37*), which belong to molecularly distinct subtypes of L2/3 neurons (*36*). The cell type positive with *Baz1a* in the visual cortex was enriched for neurons with a positive prediction error. Another study showed that *Baz1a*-positive neurons in the SSC constitutively express c-fos and exhibit high sensory feature selectivity (*38*). The L2/3 neuron subpopulation identified in this study was also enriched with neurons stably expressing c-fos and highly responding to specific types of sensory inputs. Furthermore, among the three subtypes of L2/3 neurons, *Baz1a*-positive neurons were selectively reduced in Del(1.5 Mb)/+ mice, in parallel with reduced deviance detection. Consistent with these transcriptomic results, our in situ spatial analysis demonstrated a decrease in the density of this subtype in the mutant cortex. The mean NND among these neurons was also increased in Del(1.5 Mb)/+ mice, reflecting reduced cellular density. Given that this subtype also exhibited a higher basal rate of IEG coexpression, these spatial alterations align with the attenuated ensemble activity observed in the upper-layer network. These observations collectively suggest that the L2/3 neuron subpopulation that we identified as responsible for auditory deviance detection shares the same molecular signature and functional properties as *Baz1a*-positive neurons in other sensory cortices.

The mechanism of error signal generation in the cortex has been proposed to be related to the balance between bottom-up sensory signals and top-down prediction signals (*49*, *50*). The presence of these two inputs to DD neurons has been experimentally demonstrated in the case of sensory-motor mismatch (*37*). The advantage of this experimental paradigm is that the motor output can be used as a proxy of prediction signal input to the sensory cortex. The *Baz1a*-positive neurons in this paradigm are characterized as positive prediction error neurons that receive excitatory bottom-up sensory inputs and inhibitory top-down prediction inputs (*36*). In the case of sensory-sensory mismatch, including oddball tone presentation, the functional framework of a neural circuit has not yet been established (*46*). It is not unlikely that a similar top-down prediction signal from a higher-order cortex suppresses the response to repetitive tones and the release from the prediction signal induces an enhanced error response. Mechanistically, it is conceivable that this suppression of repetitive signals is mediated by local inhibitory interneurons recruited by top-down predictions. Consequently, the prominent activity of DD neurons observed in the oddball condition could potentially reflect disinhibition arising from the absence of these predictive inhibitory signals. If this scenario holds true, then the DD neurons identified in this study may have connectivity similar to that of the positive prediction error neurons identified in the sensory-motor mismatch. This model is attractive and fits our data on DD neurons, which stably express c-fos and exhibit high functional connectivity with nearby neurons. It should be noted, however, that the functional connectivity measured here is based on correlated calcium activity and does not necessarily imply direct monosynaptic coupling.

The specific function of L2/3 neurons in AAF/A2 in the detection of sensory mismatch was selectively impaired in Del(1.5 Mb)/+ mice. We designed a tone presentation protocol as a close replicate of the method applied to humans (*18*). The population average of the calcium response recorded using two-photon imaging, together with the data obtained using wide-field imaging, showed a calcium response with a slow onset with a delay of ∼100 ms after the onset of the oddball sound. These response kinetics fit the electroencephalogram data in humans, further supporting the notion that the calcium responses from the rodent model share the same properties as the activation of the human sensory cortex. Although the error signals in the cortex were severely attenuated, the activity of thalamic afferents in Del(1.5 Mb)/+ mice was comparable to that in control mice. This observation is important, as a previous study indicated the impairment of thalamic axons in 22q11.2 deletion syndrome model mice due to presynaptic D2 receptor hyperactivation (*51*, *52*). The intact response observed in this study may reflect the developmental compensation of thalamic axons. Nevertheless, it is less likely that the impairment of thalamic axons is involved in the phenotype of Del(1.5 Mb)/+ mice in the auditory mismatch response.

MMN is one of the biomarkers with the largest effect size in patients with schizophrenia (*13*–*15*); however, animal studies on the underlying mechanisms of MMN remain scarce. Both 22q11.2 deletion syndrome and ketamine-induced schizophrenia mouse models have been shown to have functional impairments in the reliability of neuronal coactivity patterns in the visual cortex (*53*). This result is consistent with our data on the population activity of L2/3 DD neurons in the AAF/A2, which showed impaired temporal synchronization. These observations indicate that the aberrant pattern of cortical neuron ensembles is a common pathology in multiple sensory modalities of the 22q11.2 deletion syndrome mouse model (*54*). Consistently, patients with schizophrenia exhibit MMN in response to both auditory and visual stimuli (*55*). Although MMN impairments in patients are present in response to both auditory and visual stimuli, impairments in the AC may have distinct characteristics. While visual MMN has been validated in humans, several reviews have noted that visual MMN is generally weaker and more variable than auditory MMN, suggesting fundamental functions of MMN in auditory perception and cognition (*56*).

Auditory information processing is known to be both hierarchical and parallel. The hierarchical organization of the auditory pathway gradually enhances deviance detection through connections between the brain stem, medial geniculate nucleus, A1, and higher AC (*10*, *45*). In contrast, parallel pathways transmit different auditory information via the lemniscal and nonlemniscal pathways (*57*). In thalamocortical afferents, the nonlemniscal pathway responds strongly to deviant signals (*10*). Our imaging experiments on thalamic afferents in the AC confirmed these findings. Namely, the afferents in AAF/A2, which belong to the nonlemniscal pathway, showed stronger deviance detection than the afferents in A1. Regarding the hierarchical difference, we found more prominent deviance detection in AAF/A2 than in A1. Notably, significant impairment in deviance detection was present only in the postsynaptic neurons of AAF/A2 in Del(1.5 Mb)/+ mice, whereas intact error signals were present in the thalamic afferents in AAF/A2. These results indicate that either thalamocortical synapses or local AAF/A2 circuits are the circuit elements with the most prominent dysfunction in Del(1.5 Mb)/+ mice. Our snRNA-seq data support this notion, as there were significant alterations in gene expression related to synaptic structure and function in L2/3 excitatory neurons, together with reduced postsynaptic spines in c-fos (+) neurons. This transcriptomic profile is consistent with previous reports of impaired synaptic plasticity and structural deficits in 22q11.2 deletion models (*54*, *58*, *59*), suggesting that synaptic dysregulation, rather than metabolic deficits resulting from the deletion of mitochondria-related genes in the 22q11.2 region, is the primary driver of the observed circuit dysfunction. It is probable that intracortical connections between L2/3 pyramidal neurons are affected by the aberrant expression of synaptic genes, leading to reduced circuit function for the generation of locally ensembled activity. In physiological situations, this strong coactivation of L2/3 neurons is likely to send negative feedback signals to A1, as observed in this study. In our VECM analysis, this negative propagation denotes a statistical predictability where activity fluctuations in A2 lead to a delayed reduction of activity in A1. Biologically, this forms a negative feedback loop where late-phase suppression from a higher-order area regulates a lower-order area. This inhibitory connectivity aligns with the predictive coding hypothesis, in which top-down predictions suppress redundant lower-level activity, thereby enhancing the contrast of the input signal. Currently, the contribution of this retrograde negative feedback to the processing of error signals in the AC is speculative. Future functional manipulations specific to this negative feedback signal are warranted to determine its importance.

In this study, we successfully identified the critical component of deviance detection in the higher-order AC and characterized the local organization and functional connectivity of neurons that respond to mismatches. Furthermore, by identifying a selective phenotype in the DD neurons in the AC of Del(1.5 Mb)/+ mice, we provided clues to unravel the core functional alterations in MMN-generating mechanisms in animal models of psychiatric disorders. Our findings, together with those of human imaging studies on auditory mismatch, contribute to the elucidation of the fundamental mechanisms underlying auditory dysfunction in patients with schizophrenia.

## MATERIALS AND METHODS

### Experimental animals

All animal experiments were reviewed and approved by the Institutional Animal Care and Use Committee of the University of Tokyo (permission number: A2023M031-02) and conducted following “Manual for animal experiment of the University of Tokyo.” For experiments using WT mice, either CBA/CaOlaHsd or F1 hybrids generated by crossing CBA/CaOlaHsd and C57BL/6N strains were used. For experiments using disease model mice, F1 hybrids were generated by crossing WT CBA/CaOlaHsd mice with Del(1.5 Mb)/+ mice, which carry a chromosomal deletion syntenic to the 1.5-Mb deletion observed in human 22q11.2 deletion syndrome and were generated and maintained on a C57BL/6N genetic background (*60*). The light-dark cycle in the mouse breeding facility was maintained at 12/12 hours. Adult mice of both sexes, aged ≥8 weeks, were used in this study.

### Surgery for two-photon imaging of cortical cell bodies and wide-field imaging

Mice were anesthetized through the inhalation of isoflurane (0 to 3%), with the dosage adjusted to maintain an appropriate level of surgical anesthesia. To manage pain during surgery, an analgesic solution was typically prepared by dissolving 1.88 mg of medetomidine (Dorbene, Kyoritsuseiyaku, Japan), 2.00 mg of midazolam (Dormicum, Maruishi Pharmaceutical, Japan), and 2.50 mg of butorphanol (Vetorphale, Meiji Animal Health, Japan) in 50 ml of saline, administered intraperitoneally at a dose of 5 μl/g of body weight or 100 μl, and repeated as needed. The scalp was incised along the midline to expose its skull. A custom-made metal bar was attached to the skull using dental cement (Super-Bond, Sun Medical, Japan). The head of the mouse was secured on a custom-made surgical platform, with the right side positioned upward. A cranial window of ∼3 mm in diameter was made on the right temporal and parietal bones using a dental drill (VOLVERE i7 or Vmax, NSK, Japan) or a dermal punch (Maruho, Tokyo). The dura mater was then gently removed. Using a commercial motorized injector (Nanoject III, Drummond, USA or NANOLITER, World Precision Instruments, USA), the AAV solution was injected into 12 to 16 cortical sites, with volumes of 120 to 330 nl at each site. AAV9–calcium- and calmodulin-dependent protein kinase II (CamKII)–GCaMP6f–WPRE–SV40 (Addgene, USA), with a titer of 3.0 × 10^12^ to 7.5 × 10^12^ genome copies (GC)/ml, and AAV9-hsyn-RiboL1-jGCaMP8m (Addgene, USA), with a titer of 1.2 × 10^12^ to 2.3 × 10^12^ GC/ml, were injected. A double-layered coverslip [an outer coverslip with a diameter of 4 mm and thickness of no. 1 or no. 00 and an inner coverslip with a diameter of 2.8 to 3 mm and thickness of no. 4, no. 5, or 0.7 mm (Matsunami Glass, Japan)] was bonded with ultraviolet curing adhesives (NOA61 or NOA81, Norland Products, USA) and placed on the surface of the brain. A coverslip was attached to the skull using dental cement (Super-Bond, Sun Medical, Japan) or adhesive. To reverse the effects of medetomidine, an atipamezole solution (0.075 mg/ml; Kyoritsu Seiyaku, Japan) in saline was administered intraperitoneally, as needed. Mice were then returned to their home cages. We waited for an average of 9 days for GCaMP expression. AAV vectors were purchased from Penn Vector Core or produced using the ultracentrifugation method described in a previous paper (*61*).

### Surgery for two-photon imaging of thalamocortical axons

To express the genetically encoded calcium indicator GCaMP6f in thalamic axons, we locally injected AAV2/1-hsyn-GCaMP6f (final concentration: 2.07 × 10^13^ to 3.45 × 10^13^ GC/ml; University of Pennsylvania Vector Core) into the coordinates of the medial geniculate body. In some animals, the injection solution was supplemented with 9% mannitol. After the mice were anesthetized and secured in a stereotaxic frame, the scalp was incised along the midline, and a small cranial window was created using a dental drill at a position defined by the stereotaxic coordinates (3 mm posterior to the bregma and 2 mm lateral to the midline) using a dental drill. A glass micropipette filled with AAV solution was inserted at a depth of 2.5 to 3.5 mm from the dura, and 300 to 400 nl of AAV was injected. Following surgery, mice were returned to their home cages and allowed to recover. Imaging was performed 2 to 6 weeks postinjection to allow sufficient GCaMP6f expression. Cranial window surgery for in vivo imaging was performed following the same procedure as for cell body observations, except that in some animals, a double-layered glass window composed of a 4-mm-diameter no. 1 cover glass and a 2.8-mm-diameter no. 3 cover glass (Matsunami Glass, Japan) was used. Except for one animal in A1, the imaging was conducted under awake conditions.

### In vivo imaging

Wide-field imaging was performed using the fluorescence optical path of the A1 MP+ two-photon system (Nikon, Japan) with a 4× objective lens (CFI Super Fluor 4X MRF00041, Nikon). Fluorescence signals were detected using an electron-multiplying charge-coupled device camera (iXon Ultra or iXon Ultra 897, Andor Technology, Belfast, UK). Images of 128 pixels by 128 pixels were acquired at a frame rate of ∼31.5 Hz. A white light-emitting diode (SOLIS-3C, Thorlabs, NJ, USA) and fluorescence filter cube (excitation filter, 450 to 490 nm; dichroic filter, 505 nm; emission filter, 520 nm) were used.

Two-photon imaging was performed using a microscope (A1 MP+, Nikon, Japan) equipped with a water immersion objective lens [CFI75 Apochromat 25XC W 1300, Nikon, Japan; numerical aperture (NA), 1.1; working distance, 2 mm] and femtosecond laser (Maitai DeepSee, Spectra-Physics, USA). The wavelength was set to 920 nm. For cell body imaging, images of 512 pixels by 512 pixels with a field of view of 508 μm by 508 μm were acquired at a frame rate of ∼30 Hz using the NIS-Elements AR software (Nikon, Japan). L2/3 and L4/5 neurons were recorded at depths of 120 to 250 μm and 300 to 441 μm from the cortical surface, respectively. For simultaneous multiplane imaging, four planes spaced ∼17.5 to 36 μm apart were imaged at 4.9 Hz using a microscope (A1 MP+, Nikon, Japan) equipped with a piezo-driven *z*-stage. The center *z*-depth of the four planes ranged from 155 to 422 μm below the pial surface. To image thalamic axonal boutons, a field of view of 129 μm by 129 μm was acquired at a resolution of 512 pixels by × 512 pixels. Recordings were made at depths of 10 to 73 μm in L1 and 240 to 410 μm in L4, below the pial surface.

### Auditory stimulation and MMN paradigm

The head of the awake mouse was secured using a custom-made stereotaxic device to ensure that the coverslip on the right AC was positioned horizontally. All acoustic stimuli were generated using custom Python code and amplified using an amplifier (AP20d, Fostex, Japan). The sound stimuli were delivered through a speaker (FT207D, Fostex, Japan) placed 10 cm in front of the mouse. The intensity of the sound stimuli was calibrated using a sound level meter (NA-42, RION, Japan) to ensure a sound pressure level of 50 dB.

In tonotopy mapping with wide-field imaging, five pure-tone stimuli (8, 11.3, 16, 22.6, and 32 kHz) with a duration of 100 ms were presented every 30 imaging frames, resulting in an approximate stimulus onset asynchrony of 1 s. For each frequency, pure tones were presented 60 times to obtain the average tonotopy map. We implemented a frequency MMN paradigm by combining the oddball paradigm with the many-standard paradigm (*26*). In the many-standard paradigm, 10 pure tones with pitches differing logarithmically by 5% were used. In the oddball paradigm, the pitches of the third and eighth tones in 10 pitches presented in the many-standard paradigm served as the standard and oddball stimuli. The oddball stimuli were pseudo-randomly presented with 10% probability of presentation. In the many-standard paradigm, 1 of the 10 pitches was presented with 10% probability (note that this is the same presentation probability as the oddball in the oddball paradigm). The stimulus onset asynchrony was ∼1 s. Our MMN paradigm consisted of three blocks: an oddball paradigm, an oddball paradigm that reversed the pitch of the standard and oddball stimuli, and a many-standard paradigm. The evaluation of deviance detection and adaptation was based on responses to the same frequency stimuli under three different conditions, which are referred to as oddball, control, and repetitive stimuli (Fig. 2A). Oddball stimuli were rare tones in oddball blocks. Control stimuli were tones in the control paradigm with the same frequency as the repetitive and oddball tones in the oddball block. Repetitive stimuli were the last repeated tones before the rare stimuli. For multiplane two-photon imaging and wide-field imaging, calcium responses were recorded in four sessions using nonoverlapping pitches for both oddball and control stimuli. Before the sessions, spontaneous calcium activity during a 15- to 20-min period (i.e., in the absence of stimuli) was also imaged. Nonoverlapping pitches in four sessions were selected randomly from 40 pitches spaced at 5% intervals and centered at 20 kHz. The third and eighth of the 10 pitches were selected as oddball stimuli. For multiplane two-photon imaging, analyses were performed using the largest response block among the four blocks.

### Analysis of two-photon calcium imaging

Horizontal drift was corrected using either a custom script to calculate the correlation coefficient among images or the Suite2p image processing program. To isolate the fluorescence signals from cell bodies, regions of interest (ROIs) corresponding to neuronal cell bodies were extracted from the time series of images using Suite2p, with subsequent manual inspection. To isolate the cell body–derived calcium signal, we first calculated the mean fluorescence intensity in each ROI (F_roi). Next, the neuropil-derived signal was estimated from the mean fluorescence intensity of a circular area with a diameter that was six times larger than that of the cell body (F_neuropil). Last, the cell body–derived signal was estimated using the following formula

F=F_roi−0.7×F_neuropil

Axonal boutons were detected in the images after trimming the peripheral regions within 5.0 μm of the margin. This was necessary to minimize the motion artifacts. Fluorescence signals from axonal boutons exhibited large temporal fluctuations that were useful for reliably detecting the positions of axonal boutons. To detect image pixels with large temporal fluctuations, we calculated the SD of the fluorescence intensity changes for each pixel. Next we searched a pixel that exhibited the SD value that was highest within a square of 4.03 μm by 4.03 μm in the *xy* plane and that exceeded the median plus three times of SD calculated from all pixels in the analyzed image. This pixel was taken as the center of an axonal bouton, and the mean pixel intensity within a square ROI of 2.02 μm by 2.02 μm in the *xy* plane was used for fluorescence signal from a single axon. Axonal boutons identified in a single imaging area tended to contain multiple boutons en passant. To minimize the bias derived from these boutons that tended to be activated synchronously, we identified pairs of axonal boutons with temporal correlation coefficients of ≥0.5 and excluded the bouton with lower fluorescence intensity from each pair.

A 60-s moving average filter was used to remove slow fluorescence drift for each ROI of either the neuronal cell body or the axonal bouton. Fluorescence signals were divided by the median value of all frames during the session to obtain *F*/*F*_0_. The mean and SD of *F*/*F*_0_ from the time-series data were calculated, and time points exceeding the means ± 1.5 SD were excluded. To extract the baseline segment showing little calcium response, this process was repeated 15 times. The *z*-score of *F*/*F*_0_ for each cell or bouton was calculated using the SD of the remaining time points. The average response over 100 ms before the onset of the auditory stimulus was considered pre–stimulus activity. Neurons or axonal boutons were defined as responsive to tones if their average response to all stimuli in the many-standard block exceeded 3.5 SD in at least one frame within 0 to 334 ms after tone onset (i.e., covering all 300 stimuli from 10 frequencies). To ensure that the best frequency was close to the two oddball frequencies, cells or boutons that responded most strongly to the highest/lowest pitch among the 10 frequencies were excluded. For statistical analysis, oddball, control, and repetitive responses were quantified as mean values within 0 to 667 ms after stimulus onset. The response indices for the oddball, control, and repetitive conditions were defined as follows

$$\begin{array}{c} D=\sqrt{{\text{Oddball response}}^{2}+{\text{Control response}}^{2}+{\text{Repetitive response}}^{2}}\\ \text{Oddball index}=\frac{\text{Oddball response}}{D}\\ \text{Control index}=\frac{\text{Control response}}{D}\\ \text{Repetitive index}=\frac{\text{Repetitive response}}{D} \end{array}$$

For the subsets of experiments using CBA/CaOlaHsd background mice, the criteria for neurons responsive to tones were modified. Namely, neurons were considered responsive if their activity exceeded 5 SDs in at least one frame within 0 to 334 ms after tone onset in response to all stimuli in the many-standard block. This adjustment of criteria was necessary to isolate neuronal populations with comparable response rates between CBA/CaOlaHsd and F1 hybrid mice, as CBA/CaOlaHsd-background mice displayed ∼1.5-fold higher response amplitudes than F1 hybrid mice. For simultaneous multiplane imaging, the response 204 ms before the onset of an auditory stimulus was considered as the baseline activity. Neurons were defined as responsive to tones if their average response to all stimuli in the many-standard block exceeded 3.5 SD in at least one frame within 0 to 408 ms after tone onset. To ensure that the best frequency of neurons was close to the two oddball frequencies, the cells that responded most strongly to the highest/lowest pitch among the 10 frequencies were excluded. For statistical analysis, oddball, control, and repetitive responses were quantified as the mean value within 0 to 1020 ms after stimulus onset.

Although the temporal kinetics of GCaMP6f are slower than electrical spiking activity, previous studies using simultaneous electrophysiology and calcium imaging in the auditory and visual cortices have confirmed that calcium transients reliably reflect underlying neuronal firing patterns, particularly burst firing (*62*, *63*). Therefore, while our analysis does not capture millisecond-precision synchrony, the observed correlations in calcium signals serve as a robust proxy for local functional connectivity based on temporal coactivation.

### Analysis of calcium activities in neuron populations

For UMAP analysis, the response waveforms to the oddball stimulus and the preceding four repeated stimuli from both the upward and downward (i.e., inverted) oddball paradigm were averaged for each cell after applying a band-pass filter between 0.1 and 10 Hz to the *z*-scored *F* values. Neurons were included in the analysis if their average responses to all stimuli in the many-standard block exceeded 3 SDs in at least one frame within 0 to 334 ms after tone onset. Calcium responses were normalized to have a mean of 0 and SD of 1 over 150 frames, followed by dimensional reduction using the Python package of UMAP with the following settings: metric: “euclidean,” n_components: 2. The two-dimensional UMAP plot was further analyzed by overlaying a 10 × 10 grid with uniform spacing. The average calcium response in each grid was calculated to determine the trends in the transition of response patterns. DD and NS neurons were defined as those located in the region defined by [*y* < −0.6*x* + 1] (DD neurons) or [*y* > −0.6*x* + 2] (NS neurons), where *x* and *y* were the values of the UMAP dimensions 0 and 1, respectively.

To analyze the spatial distribution of cells responding to the oddball stimulus, the two-photon imaging plane was binned into 5 by 5 sections, and the number of cells within each grid square was counted. For hub neuron analysis, the correlation coefficients of calcium activity for all pairs of neurons were calculated in image sequences containing ≥10 recorded neurons. To evaluate the functional connectivity, we calculated the index of local connections of a given neuron by averaging the top 10% of the positive correlation coefficients with other cells within a distance of 50 μm in the control block. After calculating the index of local connections for all neurons in the imaging field, the values were normalized, such that the maximum and minimum values were set to 1 and 0, respectively, to obtain the LFC index. For simultaneous multiplane imaging, all positive correlation coefficients with tone-responsive neurons located within 400 μm of the spontaneous block (i.e., in the absence of tone stimulation) were included in the calculation.

The frequency-tuning bandwidth (full width at half maximum) in neurons and thalamic axon boutons was determined by fitting the responses to pure tones at each frequency in the control block using a one-dimensional Gaussian function. The bandwidth was calculated in octaves using the SD of the Gaussian fit. Only cells or boutons with an *R*^2^ value of a fit of >0.3 were included in the analysis.

The analysis of the distances between neurons was performed using imaging sequences containing at least two DD or NS cells. The mean distance between all the neuron pairs was calculated. The spatial density analysis was performed for three types of neuron groups: groups based on the best frequency, DD neurons, and NS neurons. For the best frequency group, we excluded neurons responsive to the highest and lowest of the 10 frequencies. For each neuron within a group, we calculated the density of neighboring cells in concentric 100-μm-wide rings up to a radius of 600 μm. This density was then normalized by the total cell count per field of view and subsequently averaged across all cells in the group. Similar analyses were performed for the DD and NS neurons.

### Analysis of wide-field calcium imaging

The analysis of single-photon imaging data in response to oddball paradigms was conducted by binning the raw data recorded at 128 pixels by 128 pixels into 32 pixel–by–32 pixel segments. The auditory fields for the MMN analysis were manually selected from the time-series images averaged over all auditory stimuli in both the oddball and control blocks. Because of the differences in the size of the fields, A1 was defined as a 5 pixel–by–5 pixel region, whereas AAF and A2 were defined as 3 pixel–by–3 pixel regions. To remove the effect of large signal fluctuations across the entire field of view, we first estimated the temporal fluctuation by calculating the mean fluorescence intensity of the peripheral AC, which was defined as the area of >6 pixels away from the centers of A1, AAF, and A2. The mean fluorescence signal from the peripheral AC was subtracted from the signals within the three subfields. Subsequent analysis at the auditory subregion level was performed by spatially averaging the pixel data within A1, AAF, and A2. The baseline calcium signal was defined as the mean fluorescence intensity during the 32 ms preceding the stimulus onset. The baseline was subtracted from the time series of the calcium response to obtain the net signal increase, which was further divided by the baseline to obtain the normalized calcium response. Last, it was necessary to compare responses across oddball, repetitive, and control conditions. To this end, we further divided the normalized calcium response by the square root of the sum of squares of the response within 0 to 159 ms after the stimulus onset. For the pixel-level analysis, signal transmission between multiple fields was calculated using the VECM (*43*, *64*). Specifically, after normalization with the SD during the −95- to 381-ms period relative to the tone onset (i.e., 15 frames), the Python package of VECM (Statsmodels, open source) was used with the settings of k_ar_diff = 1, seasons = 5, deterministic = n, and coint_rank = 2 during the −32- to 127-ms period relative to the tone onset (i.e., five frames). Data sequences from 30 trials, corresponding to a total of 150 frames, were concatenated. A single pixel in A1 was paired with another pixel in either AAF or A2, and the intensity values for 150 frames from the paired pixels (2 × 150 matrix) were used for model fitting. This paired calculation was performed for all possible pixel combinations. The model parameters were fitted using the maximum likelihood method, and the effects of perturbations on the model were examined using impulse-response functions. The VECM was fitted to the responses of the four frequency blocks. The transmission index was defined as the average of the values from all pixel pairs, where each pixel pair’s impulse response was assigned a value of 1 if positive and −1 if negative. To visualize the population impulse response with the relative positions of A1, AAF, and A2, the coordinate information from one sample was applied to the other samples, and the impulse response of all samples was then averaged for each pixel position. To simplify the visualization, the impulse response was plotted by averaging the pixels at the start or end points. The center of the 5 pixels by 5 pixels for A1 was set as the origin of the coordinates. The strength of the impulse is indicated by 25 arrows. The transparency of each arrow represents its relative strength, which is calculated by dividing its rank by 25. The color signifies the sign of the impulse: red for positive and blue for negative.

### Auditory brainstem response

Adult male mice were anesthetized using the analgesic solution described for cranial window surgery. Following anesthesia induction, an active electrode was placed subcutaneously on the forehead, a reference electrode was positioned below the pinna of the left ear, and a ground electrode was placed below the pinna of the right ear. Acoustic stimuli were presented in a free-field configuration using a speaker (FT28D, Fostex, Japan) positioned 10 cm from the left ear. Click stimuli (0.1-ms duration) were generated using an RZ6 workstation (Tucker-Davis Technologies, USA) and delivered with an interstimulus interval of 50 ms. The stimulus intensity was attenuated in steps of 5 to 20 dB. Auditory brainstem responses were recorded using a time-locked averaging procedure, with 500 repetitions. The recorded signals were band-pass filtered between 300 and 2000 Hz.

### Immunohistochemistry

To achieve sparse labeling of the dendrites, a mixture of AAV9-CAG-FLEX-GFP and AAV1.CamK2.0.4.Cre.SV40 (6.33 × 10^9^ GC/ml; 105558, Addgene) was injected into the right AC. A small craniotomy was carefully performed using a drill 2 mm anterior to the occipitotemporal suture, along the temporoparietal suture. A glass micropipette was inserted at a 20° angle relative to the cortical surface at a depth of 0.5 to 1.4 mm. The viral mixture was injected at eight sites (200 nl per site). Following the injection, the mice were returned to their home cages and allowed to recover for 1 week to ensure adequate GFP expression.

Mice were anesthetized and transcardially perfused with 4% paraformaldehyde in phosphate-buffered saline (PBS), followed by fixation in the same fixative. The brains were then dissected and sectioned into 100-μm-thick coronal slices using a vibratome (VT-1000S, Leica). For correlative microscopy using near-infrared branding, a laser from a two-photon microscope (A1 MP+, Nikon, Japan) was applied to some tissue samples to create the fiducial markers. Brain sections were cut at a thickness of 100 μm, in a plane parallel to the cortical surface.

The brain sections were incubated with primary antibodies at room temperature overnight, followed by incubation with secondary antibodies at room temperature for 2 or 6 hours. The antibody incubation solution contained 0.2% Triton X-100, 5% normal goat serum, and 0.01% sodium azide in PBS. The primary antibodies used in this study were rat anti–c-fos (1:200 or 1:500; Synaptic Systems), rabbit anti-GFP (1:200; MBL), and mouse anti-GAD65 (1:200; Chemicon). The secondary antibodies were Alexa Fluor 647–conjugated goat anti-rat immunoglobulin G (IgG; 1:200; Invitrogen), Alexa Fluor 546–conjugated goat anti-rat IgG (1:500; Invitrogen), Alexa Fluor 488–conjugated goat anti-rabbit IgG (1:200; Invitrogen), and Alexa Fluor 546–conjugated goat anti-mouse IgG (1:200; Invitrogen). Hoechst 33342 (1:500; Fujifilm) was used to visualize nuclei.

### Imaging of fixed samples

Following immunostaining, the brain sections were mounted in either PBS or 70% glycerol in PBS and imaged using a confocal laser-scanning microscope (A1R, Nikon). Imaging of dendritic structures and GAD65 puncta was performed using a 40× silicone immersion lens (Plan Apo, Nikon; NA, 1.25) with a confocal aperture set to 1.0 airy unit. Imaging of c-fos–labeled cells was performed using a 20× lens (Plan Apo, Nikon; NA, 0.75) or 10× lens (Plan Apo, Nikon; NA, 0.45). All images were acquired from the AC. Imaging of dendritic spines and GAD65 puncta was performed with a field of view of 39.61 μm by 39.61 μm, acquiring image stacks with 1024 pixels by 1024 pixels in the *xy* plane. Correlative confocal microscopy after in vivo two-photon imaging was performed at a pixel size of 1.24 μm per pixel.

In vivo two-photon images and confocal microscopic images were aligned by the GCaMP channel using the Moving Least Squares plug-in in Fiji, based on manually marked landmarks, such as bright cells and blood vessel branching points. The immunoreactivity of c-fos was quantified by calculating the mean pixel value within the ROI positioned in the GCaMP-expressing cell body. The relative c-fos intensity was calculated by dividing the target signal by the mean of the bottom 5% intensity of c-fos–positive neurons, which was considered as the signal of c-fos–negative neurons.

For dendritic spine density analysis, only dendrites from cells with somata located within 350 μm of the pia were included. Basal dendrites distal to the first branch points were randomly imaged, and oblique dendrites were included. Spine density was quantified by counting protrusions extending laterally (in the *xy* plane) from the dendritic shaft, using both maximum projection images and original *z*-stack images acquired at 0.5-μm steps in the *z* direction.

To analyze the number and area of GAD65-positive puncta around the soma, we only selected somata within 350 μm of the pia. The number of GAD65-positive puncta per soma was counted in a single *z*-plane image per cell. The area of the puncta was manually traced using the Fiji software.

For the Sholl analysis, only cells with somata located within 350 μm of the pia were analyzed. Imaging was performed at 1-μm intervals in the rostrocaudal direction as the *z* axis, with the *xy*-imaging planes centered at the soma and their sizes of 317 μm by 317 μm. When the imaged cell body was positioned <15 μm from the sample surface, the data were discarded. All basal dendrites were traced using both the maximum projection images and the original three-dimensional stack images. The number of intersections between the dendrites and concentric spheres centered on the soma with radii of 20, 40, 60, 80, 120, and 140 μm was counted.

To analyze the c-fos/GFP intensity in the soma, we divided the mean intensity of the c-fos channel by the mean intensity of the GFP channel for each cell body using a single *z*-plane image per cell.

### Sample collection for snRNA-seq

Four 19-week-old male mice per genotype were euthanized via cervical dislocation, and their brains were dissected. On the basis of brain stereotaxic coordinates, tissue samples were collected from the right hemisphere using a 2-mm-diameter dermal punch centered on the PFC [anteroposterior (AP), 2.0 mm; mediolateral (ML), 1.1 mm; from bregma], AC (AP, −2.9 mm; ML, 4.1 mm), and SSC (AP, −1.5 mm; ML, 2.5 mm). The collected tissues were immediately stored at −80°C until further analysis.

### snRNA-seq data processing

The punched tissue samples were collected into six tubes for snRNA-seq libraries (library #1: AC hetero, *n* = 4; #2: AC WT, *n* = 4; #3: PFC hetero, *n* = 4; #4: PFC WT, *n* = 4; #5: SSC hetero, *n* = 4; #6: SSC WT, *n* = 4). Punched tissues were homogenized using the Minute Detergent-Free Single Nuclei Isolation Kit (Invent Biotechnologies Inc.) to isolate nuclei according to the manufacturer’s protocol, resuspended in 100 μl of nuclei wash and resuspension buffer containing 4′,6-diamidino-2-phenylindole, and then filtered with 40-μm cell strainers.

The isolated cell nuclei were sorted using 4′,6-diamidino-2-phenylindole fluorescence with a cell sorter (SH800, Sony). Following the manufacturer’s protocol, 10× chromium libraries were prepared using the Chromium NEXT GEM Single Cell Library Kit v3.1 (PN-1000269, 10x Genomics). cDNA with cell barcode identifiers was polymerase chain reaction amplified, and sequencing libraries were prepared. The constructed library was sequenced on the Illumina NovaSeq X Plus platform. Cell Ranger Software (v7.1.0) was used for barcode processing, single-cell 3′ unique molecular identifier (UMI) counting, and mapping of the mouse genome. The mouse genome (Mus_musculus.GRCm39.107) was used as a mapping reference genome.

### snRNA-seq data analysis

Further analysis was conducted with Scanpy (v1.10.1) and AnnData (v0.10.7). Low-quality nuclei were excluded by unsupervised clustering using the Leiden algorithm (*65*), with the parameter set to a resolution of 1.0, followed by manual inspection. Cells with total UMI counts in the top or bottom 10% within each batch and those with mitochondrial UMI counts exceeding 2% of the total were excluded. Doublets were removed using Scrublet with default settings (*66*). UMIs per nucleus were normalized to 10,000, followed by natural logarithm transformation using log1p. For annotation, CellTypist (*67*) was used with default settings. The model “Mouse_Isocortex_Hippocampus” was applied. Batch correction was performed using a method based on the original Harmony procedure (*68*). Specifically, the top 2000 highly variable genes were selected across all nuclei. The data were then scaled to unit variance and zero mean, with values capped at a maximum of 10 to minimize the influence of extreme outliers. Subsequently, the principal components analysis was performed. To correct for batch effects, the harmony_integrate function implemented in Scanpy was applied.

The nuclear gene expression profile was visualized by embedding it into UMAP (*28*) using the top 50 principal components, with the parameters set to min_dist = 0.5, spread = 1.0, and n_neighbors = 15, and using the Euclidean distance metric. For the DEG analysis, nuclei with at least 4000 total UMIs and genes detected in at least 10,000 nuclei were included to ensure robustness. To identify DEGs between genotypes, statistical testing was performed using the Welch’s *t* test. Multiple testing corrections were applied using the Benjamini-Hochberg FDR adjustment to control for false positives. DEGs were defined as genes with an absolute log_2_ fold change of >0.3 and an FDR of <0.01. For visualization in volcano plots, *P* values of 0 were set to 10 to 300. GO term enrichment analysis was performed using DEGs, and g:Profiler was used with the parameters “significance_threshold_method”: “FDR,” “organism”: “mmusculus,” and “sources”: “GO:CC” (*69*).

To analyze the L2/3 IT CTX subtypes, unsupervised clustering was performed using the Leiden algorithm (*65*). The expression of the marker genes *Baz1a*, *Adamts2*, and *Agmat*, which have been previously reported (*36*, *38*, *70*), was *z*-scored across cells and then averaged across genes. The *Baz1a* marker gene set included *Tnfaip6*, *Adcyap1*, *Ptgs2*, *Pim1*, *Nefl*, *Cdkn1a*, *Tiparp*, *Rrad*, *Egr4*, *Diaph3*, *Arc*, *Fosl2*, *Egr2*, *Baz1a*, *Mas1*, *Nptx2*, *Phf21b*, *Fosb*, *Gpr3*, *Shroom3*, *Fos*, *Klf5*, and *Rnd3*. The *Adamts2* marker gene set included *Ptpru*, *Rhbdl3*, *Timp2*, *Cdh13*, *Wnt10a*, *Zfp811*, *Pea15a*, *Cbr3*, *Adamts2*, *Pde9a*, *Rfx3*, *Raver2*, *Tox3*, *Matn2*, *Syt10*, *Ngb*, *Cpne7*, *Dpyd*, *Gsta4*, and *Col23a1*. The *Agmat* marker gene set included *Maf*, *Slc24a2*, *Coch*, *Medag*, *Cxcl12*, *Etnk2*, *Cdh12*, *Rasgrp1*, *Agmat*, *Sorcs1*, *Penk*, *Lynx1*, *Serinc2*, *Ccnd1*, *S100a6*, *Abracl*, Igfn1, *Serpinb8*, *Adam33*, *Scn1b*, and *Plekha4*. The cell subtypes were assigned on the basis of the highest mean *z*-score calculated for the three gene sets. Last, normalization was performed to ensure that the total number of cells in the three subtypes was equal between batches of single-nucleus samples.

### Spatial transcriptomics

Mice were euthanized, and the brains were rapidly harvested. The brains were embedded in OCT compound (Tissue-Tek 4583, Sakura Finetek, Japan) and rapidly frozen by immersing the mold in isopentane (166-00615, Wako, Japan), chilled on dry ice. The samples were stored at −80°C and sectioned at 10 μm. Coronal sections containing the AC were identified on the basis of stereotaxic coordinates (AP axis, −2.9 mm from bregma), and the anatomical structures were shown in the mouse brain atlas (*71*).

Spatial transcriptomic analysis was performed using the Xenium In Situ Gene Expression platform (10x Genomics, USA). Tissue sections were mounted onto Xenium slides. In addition to the original gene set included in the Xenium Mouse Brain Gene Expression Panel, genes of interest were added using a custom Xenium panel. Probe hybridization, ligation, amplification, and autofluorescence quenching were performed according to the manufacturer’s instructions. For automated cell segmentation, tissues were stained using Xenium In Situ Cell Segmentation reagents with a multitissue stain mix to enable automated identification of cell boundaries. This multitissue stain mix includes antibodies for membrane labeling, antibodies for labeling cell interiors, a universal ribosomal RNA label, and 4′,6-diamidino-2-phenylindole for nuclear staining. Last, the slides were processed on the Xenium Analyzer for imaging and decoding according to the manufacturer’s workflow.

Analyses were performed by modifying the pipeline originally developed for snRNA-seq for application to Xenium data, as follows. The auditory cortical region was defined as the portion of the bilateral 0° to 30°sectors, centered on the mean coordinate of all cells (which approximately corresponded to the center of the cerebral aqueduct), that overlapped with the cortex. Unsupervised clustering was performed using the Leiden algorithm (*65*). The cluster containing the largest number of cells annotated as “L2/3 IT CTX” by Celltypist (*67*) was automatically defined as the L2/3 IT CTX cluster. The area occupied by L2/3 IT CTX cells within the AC was calculated by performing Delaunay triangulation on the cell coordinates and summing the areas of the resulting triangles. Among L2/3 IT CTX cells, the *Baz1a*, *Agmat*, and *Adamts2* subtypes were identified using the following marker genes, which represent a subset of the gene lists used in our snRNA-seq analysis. *Baz1a*: *Arc*, *Baz1a*, *Egr4*, *Fos*, *Fosl2*, *Mas1*, *Nefl*, *Nptx2*, *Phf21b*, *Ptgs*2, and *Tiparp*; *Adamts2*: *Cdh13*, *Col23a1*, *Dpyd*, *Rfx3*, *Tox3*, *Zfp811*, *Adamts2*, *Pea15a*, *Matn2*, *Raver2*, *Timp2*, and *Rhbdl3*; *Agmat*: *Agmat*, *Igfn1*, *Maf*, *Penk*, *S100a6*, *Coch*, *Serinc2*, *Etnk2*, *Adam33*, *Serpinb8*, and *Ccnd1*. After unsupervised clustering with the Leiden algorithm (*65*), each cluster was assigned to the subtype with the highest mean *z*-score–normalized expression. IEG-positive expression was defined as the proportion of cells with a *z*-score greater than 3.

### Statistical analysis

Statistical analyses were performed using Python and SciPy. Student’s *t* tests, Welch’s *t* test, Mann-Whitney *U* test, ANOVA, and a linear mixed-effects model were used with Bonferroni corrections and FDR adjustments applied for multiple testing when necessary. All tests were two tailed. No statistical tests were performed to determine the sample sizes. Data are presented as the means ± SE, unless otherwise specified. *P* < 0.05 was considered statistically significant (*P < 0.05, **P < 0.01, ***P < 0.001, and ****P < 0.0001).

## Supplementary Materials

### This PDF file includes:

Figs. S1 to S5 Supplementary Data for Statistical Analysis
