# Supplementary material for: Strong connections of locally clustered neurons in the upper-layer cortex underlie schizophrenia-related auditory deviance detection
Source: Sci Adv. 2026 Aug 14;12(33):eaeb4666. doi: 10.1126/sciadv.aeb4666 (PMC13475605; doi:10.1126/sciadv.aeb4666)
Supplement: Supplementary file 1 [file sciadv.aeb4666_sm.pdf]

Science Advances
AAAS

Supplementary Materials for

Strong connections of locally clustered neurons in the upper-layer cortex underlie schizophrenia-related auditory deviance detection

Shunsuke Mizutani *et al.*

Corresponding author: Shigeo Okabe, shigeo.okabe@riken.jp

Sci. Adv. **12**, eaeb4666 (2026)
DOI: 10.1126/sciadv.aeb4666

This PDF file includes:

Figs. S1 to S5
Supplementary Data for Statistical Analysis

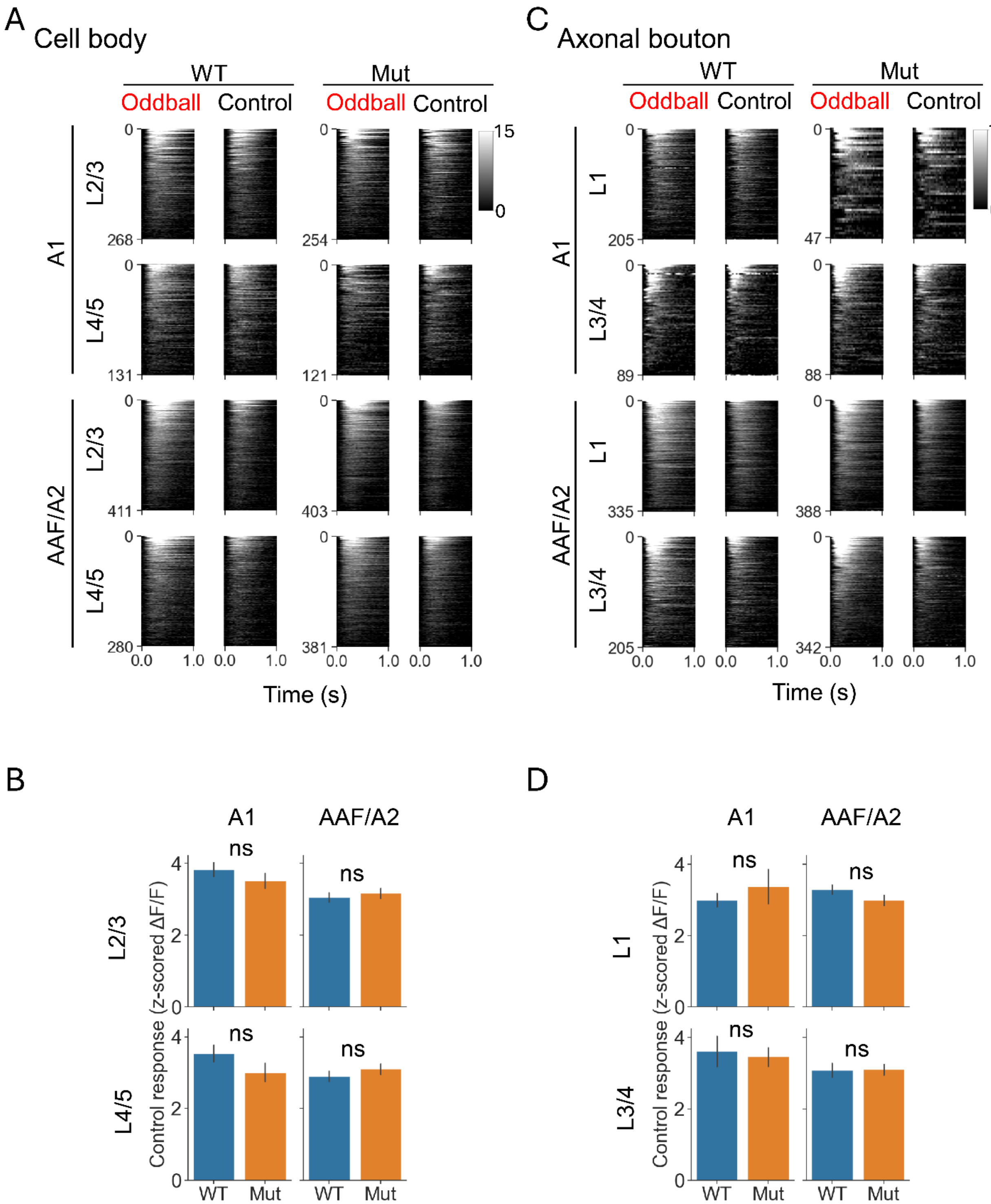

A Cell body
WT
Mut
Oddball
Control
A1
L2/3
L4/5
AAF/A2
Time (s)
C Axonal bouton
L1
L3/4
B
D
Control response (z-scored ΔF/F)
ns

Figure S1. Calcium responses of individual neurons and thalamocortical axonal boutons.

(A) Calcium responses of individual neurons 0–1 s after the presentation of oddball or control stimuli. Individual neuronal responses were aligned vertically in the order of the mean response amplitudes. The responses of L2/3 and L4/5 neurons in primary AC (A1) and anterior auditory field (AAF)/secondary AC (A2) are presented.

(B) Mean calcium responses from 0 to 667 ms after presentation of control stimuli. Wild-type (WT) and mutant (Mut) neurons in L2/3 (top) and L4/5 (bottom) of A1 (left) and AAF/A2 (right) are shown (For A and B: WT neurons analyzed, A1 L2/3: n = 269, A1 L4/5: n = 132, AAF/A2 L2/3: n = 412, AAF/A2 L4/5: n = 281; Mut neurons analyzed: A1 L2/3: n = 255, A1 L4/5: n = 122, AAF/A2 L2/3: n = 404, AAF/A2 L4/5: n = 382).

(C) Calcium responses of thalamocortical axonal boutons 0–1 s after presentation of oddball or control stimuli. Individual responses were aligned vertically in the order of the mean response amplitudes. The responses of L1 and L3/4 boutons in A1 and AAF/A2 are presented.

(D) Mean calcium responses from 0 to 667 ms after the presentation of control stimuli. WT and Mut axonal boutons in L1 (top) and L3/4 (bottom) of A1 (left) and AAF/A2 (right) are shown (For C and D: WT boutons analyzed, A1 L1: n = 206, A1 L3/4: n = 90, AAF/A2 L1: n = 336, AAF/A2 L3/4: n = 206; Mut boutons analyzed, A1 L1: n = 48, A1 L3/4: n = 89, AAF/A2 L1: n = 389, AAF/A2 L3/4: n = 343).

Data are presented as mean ± standard error of mean (SEM). n.s.; not statistically significant, Bonferroni-corrected Student's *t*-test.

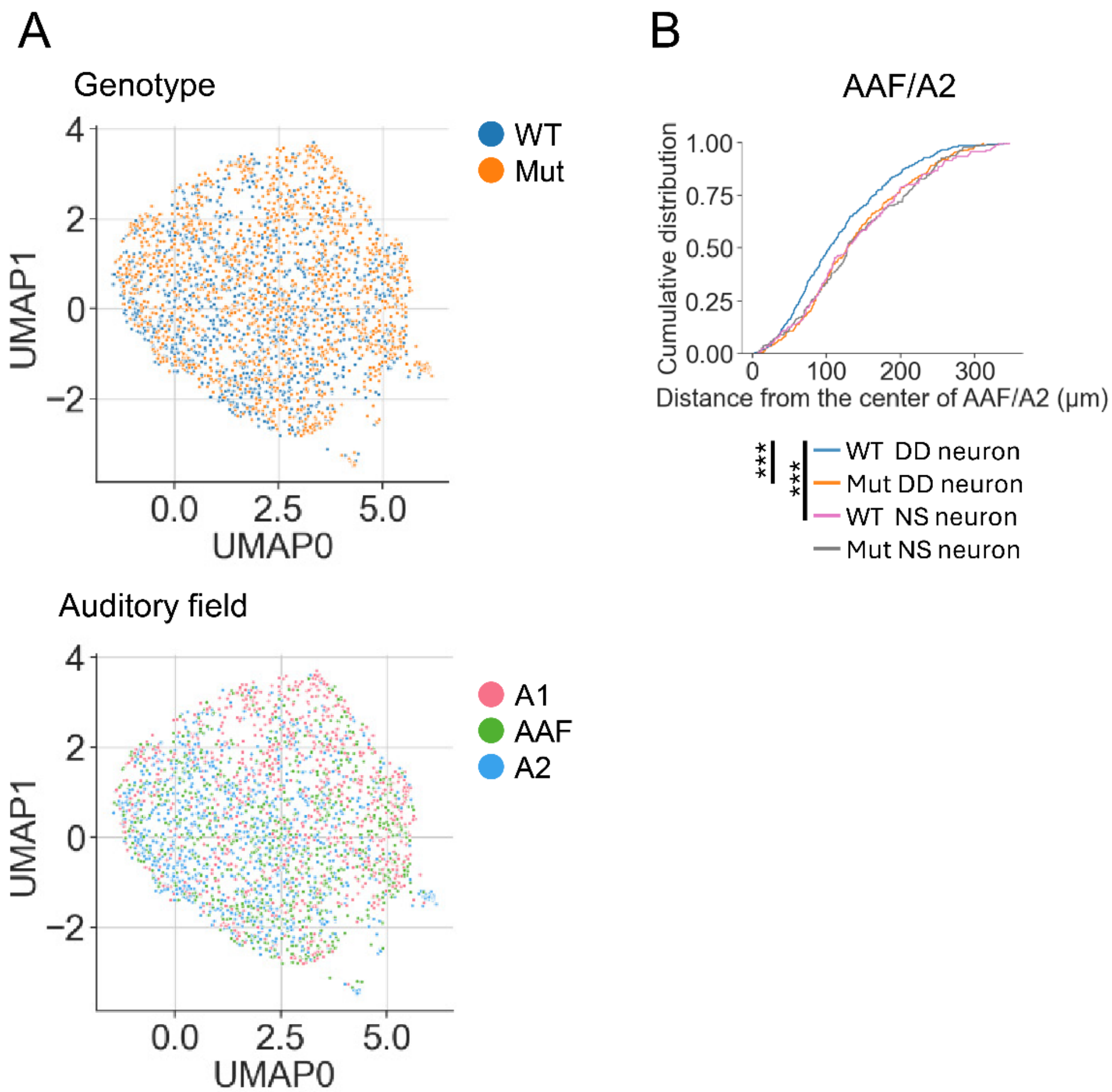
A
Genotype
WT
Mut
UMAP1
UMAP0
4
2
0
−2
0.0
2.5
5.0
Auditory field
A1
AAF
A2
UMAP1
UMAP0
B
AAF/A2
Cumulative distribution
1.00
0.75
0.50
0.25
0.00
0
100
200
300
Distance from the center of AAF/A2 (μm)
***
***
WT DD neuron
Mut DD neuron
WT NS neuron
Mut NS neuron

Figure S2. UMAP projections and spatial distribution of DD and NS neurons.

(A) Neurons are plotted on the uniform manifold approximation and projection (UMAP) based on their genotypes (top) and auditory fields (bottom). The analysis is based on data from 1,428 WT neurons and 1,518 Mut neurons.

(B) Cumulative frequency distribution of the distances between the center of the AAF/A2 and L2/3 pyramidal neuron positions. The profiles of deviance-detecting (DD) and non-selective (NS) neurons in WT and Mut mice are presented (WT DD, n = 280 cells; Mut DD, n = 225 cells; WT NS, n = 166 cells; Mut NS, n = 191 cells).

***$p < 0.001$, Student's t-test.

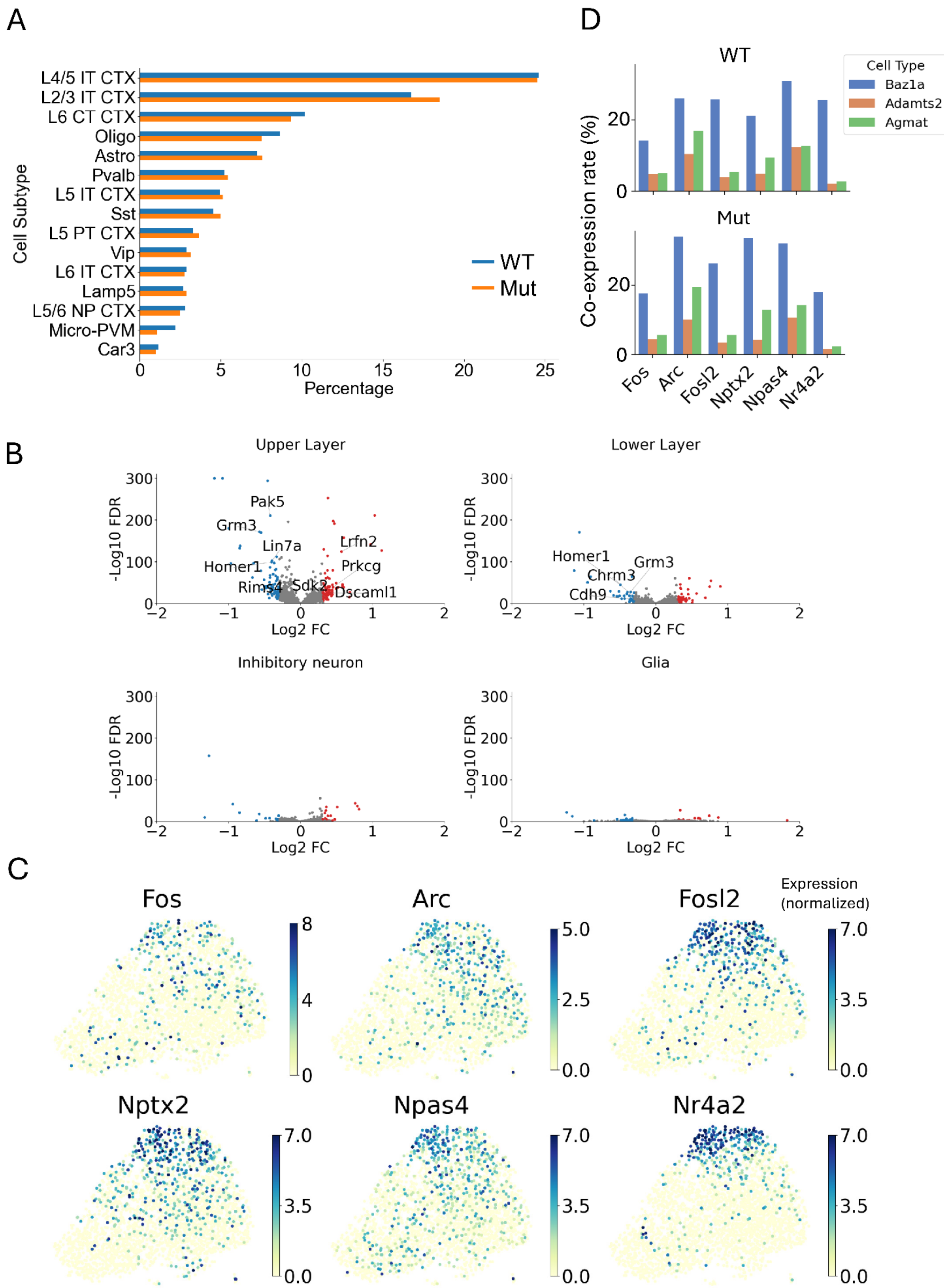

A
L4/5 IT CTX
L2/3 IT CTX
L6 CT CTX
Oligo
Astro
Pvalb
L5 IT CTX
Sst
L5 PT CTX
Vip
L6 IT CTX
Lamp5
L5/6 NP CTX
Micro-PVM
Car3
Cell Subtype
Percentage
WT
Mut
D
WT
Mut
Co-expression rate (%)
Cell Type
Baz1a
Adamts2
Agmat
Fos
Arc
Fosl2
Nptx2
Npas4
Nr4a2
B
Upper Layer
Lower Layer
Inhibitory neuron
Glia
-Log10 FDR
Log2 FC
Pak5
Grm3
Lin7a
Lrfn2
Homer1
Prkcg
Rims4
Sdk2
Dscaml1
Homer1
Chrm3
Grm3
Cdh9
C
Fos
Arc
Fosl2
Nptx2
Npas4
Nr4a2
Expression (normalized)

Figure S3. Proportion of cell types, differentially expressed genes, and IEG expression in AC neurons.

(A) Proportion of cell types in WT and Mut mice. Of the 29 annotated cell clusters, 15 clusters with cell numbers ≥ 1% of the total population were selected.

(B) Volcano plots showing differentially expressed genes for excitatory neurons in the upper layer (top left), excitatory neurons in the lower layer (top right), inhibitory neurons (bottom left), and glial cells (bottom right). Genes shown in red have a log2 fold change > 0.3 and false discovery rate (FDR) < 0.01, while genes shown in blue have a log2 fold change < −0.3 and FDR < 0.01, both of which are significantly different in their expression between genotypes. A subset of synapse-related genes with significant differences in expression is labeled by their names.

(C) Enrichment of neurons with high expression of immediate-early genes (IEGs) (*Fos*, *Arc*, *Fosl2*, *Nptx2*, *Npas4*, and *Nr4a2*) in the upper part of the L2/3 intratelencephalic (IT) cortical (CTX) neuron cluster. The overall spatial patterns of neurons with high IEGs correspond to those of Baz1a-positive neurons, as shown in Figure 5h. The color bars indicate the expression levels of the IEGs.

(D) The proportion of neurons positive for IEGs (*Fos, Arc, Fosl2, Nptx2, Npas4, and Nr4a2*) was calculated for each of the three L2/3 IT neuron subtypes (Baz1a, Adamts2, and Agmat). Positive IEG expression was defined as a transcript count of ≥1.

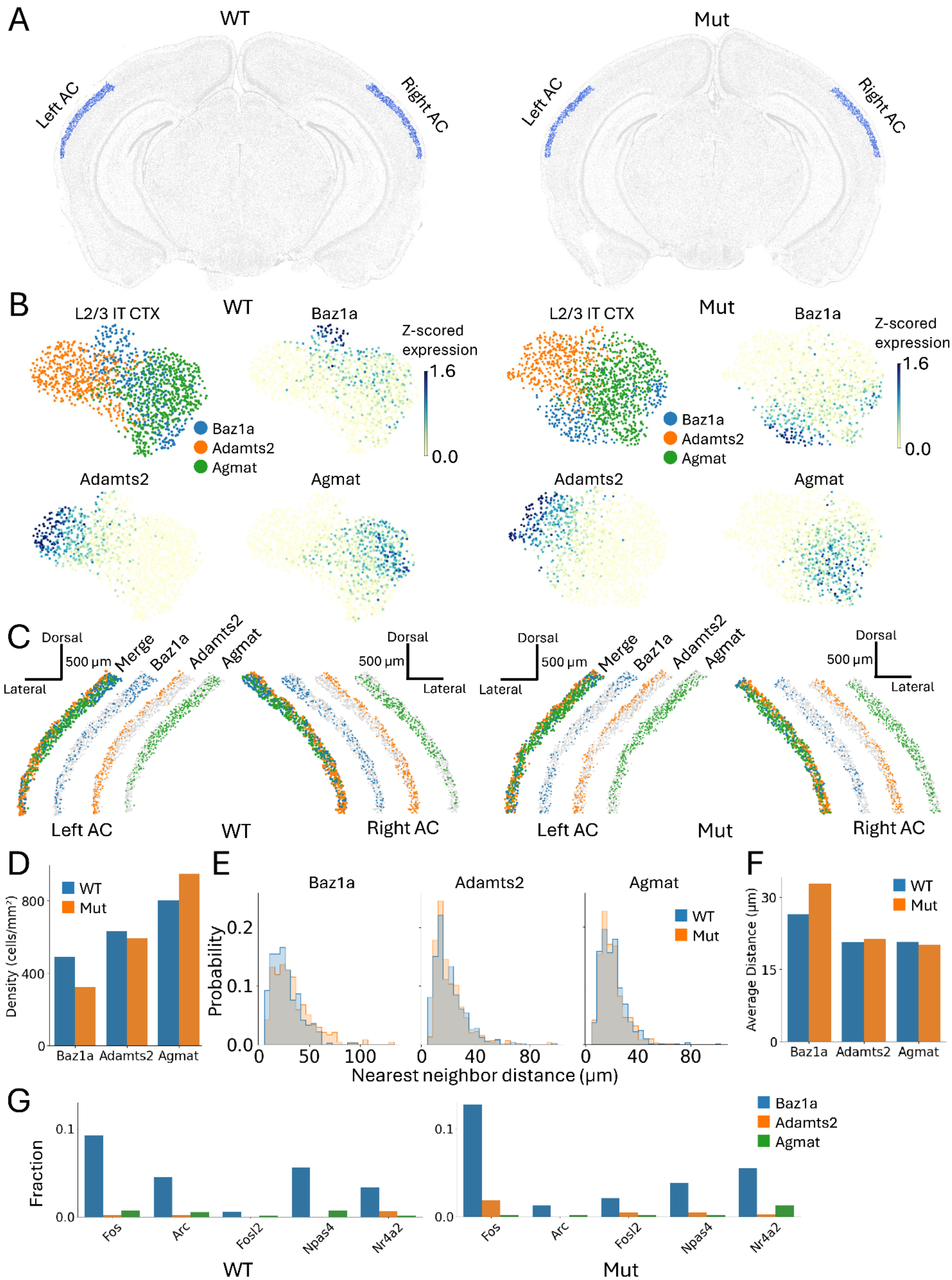
A
WT
Mut
Left AC
Right AC
B
L2/3 IT CTX
Baz1a
Adamts2
Agmat
Z-scored expression
1.6
0.0
C
Dorsal
Lateral
500 µm
Merge
Left AC
Right AC
D
Density (cells/mm²)
E
Probability
Nearest neighbor distance (µm)
F
Average Distance (µm)
G
Fraction
Fos
Arc
Fosl2
Npas4
Nr4a2

Figure S4. Spatial transcriptomics of L2/3 IT CTX subtypes in the AC.

(A) Spatial distribution of all detected cells (gray) and L2/3 cells (blue) within the AC of wild-type (WT, left) and mutant (Mut, right) mice, acquired using the Xenium platform.

(B) Subtype classification (top left) of L2/3 IT CTX neurons into Baz1a (blue), Adamts2 (orange), and Agmat (green) populations within the AC of WT (left) and Mut (right). Average expression levels of the respective marker genes are shown for Baz1a (top right), Adamts2 (bottom left), and Agmat (bottom right). The number of cells per subtype is as follows: WT: Baz1a (*n* = 356), Adamts2 (*n* = 457), Agmat (*n* = 581); Mut: Baz1a (*n* = 235), Adamts2 (*n* = 432), Agmat (*n* = 692) (**B–F**).

(C) Spatial organization of the Baz1a (blue), Adamts2 (orange), and Agmat (green) L2/3 IT CTX subtypes in the AC of WT (left) and Mut (right) mice, corresponding to the left and right sides of the auditory cortices indicated in (A).

(D) Spatial densities of the three L2/3 IT CTX neuronal subtypes in the AC of WT (blue) and Mut (orange) mice.

(E) Nearest-neighbor distances among cells of the same L2/3 IT CTX subtype in the AC of WT (blue) and Mut (orange) mice.

(F) Mean nearest-neighbor distances for each of the three L2/3 IT CTX subtypes in the AC of WT (blue) and Mut (orange) mice, calculated from the data in (E).

(G) Proportion of cells with IEG expression z-scores > 3 among Baz1a (blue), Adamts2 (orange), and Agmat (green) L2/3 IT CTX subtypes in the AC of WT (left) and Mut (right) mice.

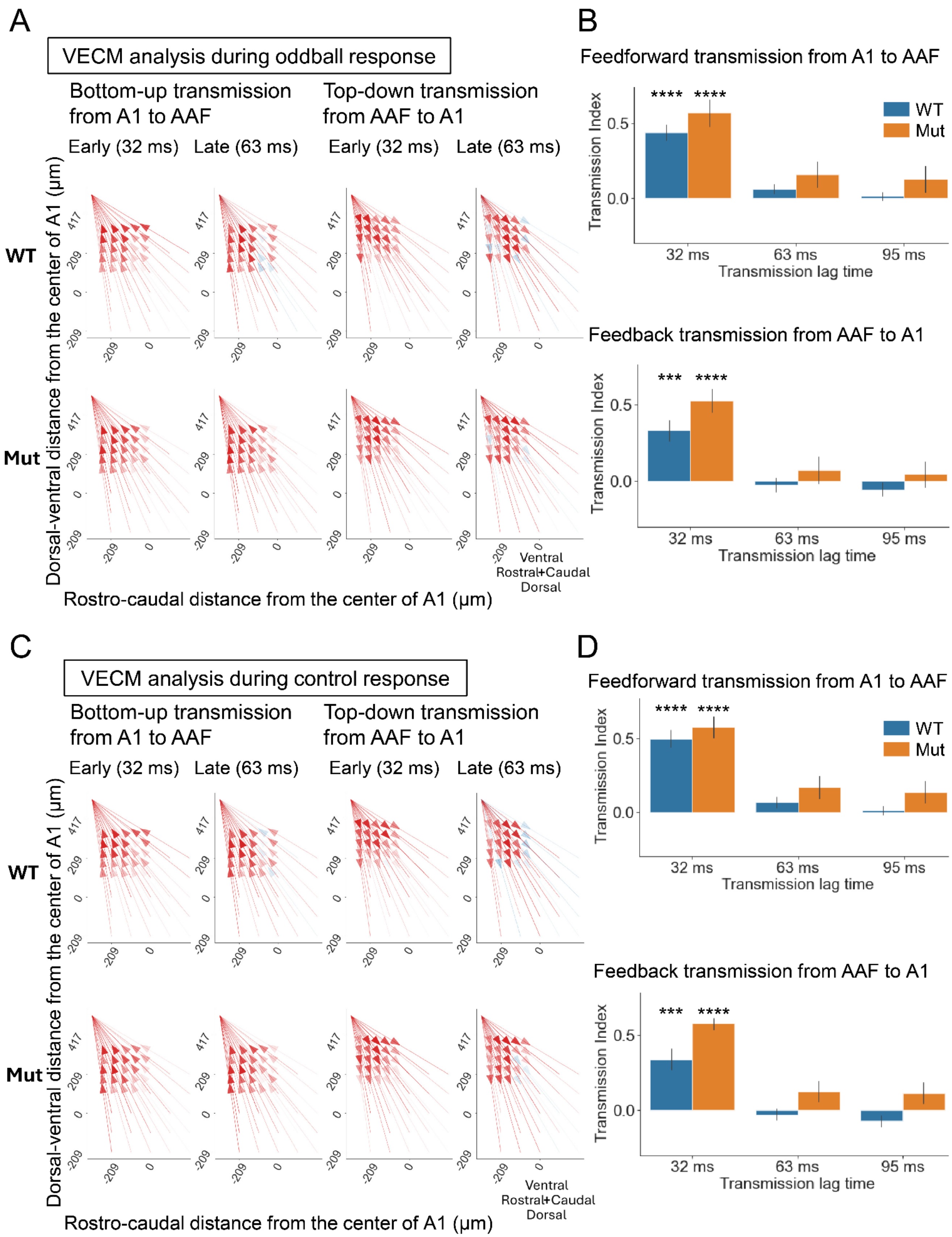

A
VECM analysis during oddball response
Bottom-up transmission from A1 to AAF
Top-down transmission from AAF to A1
Early (32 ms)
Late (63 ms)
Early (32 ms)
Late (63 ms)
WT
Mut
Dorsal-ventral distance from the center of A1 (μm)
Rostro-caudal distance from the center of A1 (μm)
417
209
0
-209
Ventral
Rostral+Caudal
Dorsal
B
Feedforward transmission from A1 to AAF
Feedback transmission from AAF to A1
Transmission Index
Transmission lag time
32 ms
63 ms
95 ms
WT
Mut
****
***
C
VECM analysis during control response
D

Figure S5. Feedforward and feedback signal transmission between A1 and AAF.

(A) Feedforward signal transmission from A1 to AAF (left) and feedback signal transmission from AAF to A1 (right) evoked by oddball stimuli in WT (top) and Mut (bottom) mice, with early (32 ms) and late (63 ms) signal transmission latencies. The direction of the arrowheads on the 5 × 5 subregions of A1 indicates whether the signals are feedforward or feedback. The intensities of the arrowheads and lines indicate signal size. The colors of arrowheads and lines indicate positive (red) or negative (blue) effects on the target areas.

(B) Extent of signal transmission (transmission index) in the feedforward (top) and feedback (bottom) directions between A1 and AAF, with transmission latencies of 32 (left), 63 (mid), and 95 ms (right) in response to oddball stimuli.

(C) Results of the vector error correction model (VECM) analysis similar to (A) for the response evoked by the control stimuli.

(D) Mean transmission index similar to (B) for the response evoked by the control stimuli (n = 20 sessions from five WT mice and 16 sessions from four Mut mice).

Data are presented as mean ± SEM. ***$p < 0.001$, ****$p < 0.0001$, Student's t-test.

Supplementary Data for Statistical Analysis

Figure 1 (E)

Frequency profiles of mean bouton responses;

WT boutons analyzed, A1 L1: n = 149, A1 L3/4: n = 69, AAF/A2 L1: n = 202, AAF/A2 L3/4: n = 137

Mut boutons analyzed, A1 L1: n = 35, A1 L3/4: n = 65, AAF/A2 L1: n = 234, AAF/A2 L3/4: n = 185

BWHM calculated by Gaussian fitting of the mean bouton responses;

WT boutons analyzed, A1 L1: n = 120 boutons from 6 animals, A1 L3/4: n = 53 boutons from 5 animals, AAF/A2 L1: n = 146 boutons from 7 animals, AAF/A2 L3/4: n = 108 boutons from 6 animals

Mut boutons analyzed, A1 L1: n = 28 boutons from 3 animals, A1 L3/4: n = 55 boutons from 3 animals, AAF/A2 L1: n = 180 boutons from 6 animals, AAF/A2 L3/4: n = 133 boutons from 6 animals

Figure 1 (J)

Frequency profiles of mean neuronal responses;

WT neurons analyzed, A1 L2/3: n = 234, A1 L4/5: n = 107, AAF/A2 L2/3: n = 245, AAF/A2 L4/5: n = 179

Mut neurons analyzed, A1 L2/3: n = 228, A1 L4/5: n = 105, AAF/A2 L2/3: n = 244, AAF/A2 L4/5: n = 259

BWHM calculated using Gaussian fitting;

WT neurons analyzed, A1 L2/3: n = 212 neurons from 5 animals, A1 L4/5: n = 96 neurons from 5 animals, AAF/A2 L2/3: n = 209 neurons from 6 animals, AAF/A2 L4/5: n = 139 neurons from 5 animals

Mut neurons analyzed, A1 L2/3: n = 212 neurons from 6 animals, A1 L4/5: n = 101 neurons from 5 animals, AAF/A2 L2/3: n = 201 neurons from 6 animals, AAF/A2 L4/5: n = 207 neurons from 6 animals

Figure 2 (D)

Response to an oddball stimulus;

n = 20 sessions from five WT mice and 16 sessions from four Mut mice.

Figure 2 (E)

Single-neuron calcium responses to control and oddball stimuli;

n = 1094 cells for WT, *n* = 1163 cells for Mut.

Figure 2 (F and G)

Single neuron response to oddball and control stimuli;

Number of WT neurons analyzed, A1 L2/3: *n* = 269, A1 L4/5: *n* = 132, AAF/A2 L2/3: *n* = 412, AAF/A2 L4/5: *n* = 281

Number of Mut neurons analyzed, A1 L2/3: *n* = 255, A1 L4/5: *n* = 122, AAF/A2 L2/3: *n* = 404, AAF/A2 L4/5: *n* = 382

Figure 2 (H and I)

Single thalamocortical axonal bouton response to oddball and control stimuli:

Number of WT boutons analyzed, A1 L1: *n* = 206, A1 L3/4: *n* = 90, AAF/A2 L1: *n* = 336, AAF/A2 L3/4: *n* = 206

Number of Mut boutons analyzed, A1 L1: *n* = 48, A1 L3/4: *n* = 89, AAF/A2 L1: *n* = 389, and AAF/A2 L3/4: *n* = 343

Figure 3 (H)

Mean pairwise distance of DD and NS neurons;

Field of views [FOVs] analyzed for DD neurons, A1 WT: *n* = 21, A1 Mut: *n* = 16, AAF/A2 WT: *n* = 45, AAF/A2 Mut: *n* = 44

FOVs analyzed for NS neurons, A1 WT: *n* = 25, A1 Mut: *n* = 28, AAF/A2 WT: *n* = 36, AAF/A2 Mut: *n* = 32

Figure 3 (J and K)

Spatial distribution of DD, NS, and frequency-responsive neurons in AAF/A2;

AAF/A2 WT: *n* = 75 FOVs, AAF/A2 Mut: *n* = 63 FOVs for best frequency neurons

AAF/A2 WT: *n* = 45 FOVs, AAF/A2 Mut: *n* = 44 FOVs for DD neurons

AAF/A2 WT: *n* = 36 FOVs, AAF/A2 Mut: *n* = 32 FOVs for NS neurons

Figure 4 (A and B)

Local functional connection index of neurons in WT and Mut mice;

L2/3 pyramidal neurons in the AAF/A2, *n* = 368 for WT, *n* = 340 for Mut

Figure 4 (C)

L2/3 pyramidal neurons in the AAF/A2:

bottom 33% of WT, *n* = 138; top 33% of WT, *n* = 103

bottom 33% of Mut, *n* = 118; top 33% of Mut, *n* = 113

Figure 4 (D)

Relationship between the LFC index and deviance detection index (L2/3 pyramidal neurons in the AAF/A2);

n = 368 for WT and *n* = 340 for Mut

Figure 4 (E)

Relationship between deviance detection index and LFC index (L2/3 pyramidal neurons in the AAF/A2);

n = 138, 127, and 103 for the top, middle, and bottom 33% of WT neurons, respectively

n = 113, 109, and 118 for the top, middle, and bottom 33% of Mut neurons, respectively

Figure 4 (F)

Relationship between LFC index, deviance detection index, and adaptation index (pyramidal neurons in the AAF/A2):

n = 800 neurons.

Figure 4 (G)

Relationship between LFC index without tone presentation and deviance detection index (pyramidal neurons in the AAF/A2):

n = 267, 266, and 267 for the top, middle, and bottom 33% of WT neurons, respectively.

Figure 5 (I)

Proportions of WT and Mut neurons for *Baz1a*+ and *Baz1a*− neurons and regions; Number of cells analyzed in the AC:

WT, *Baz1a*+ = 323, *Baz1a*− = 897; Mut, *Baz1a*+ = 268, *Baz1a*− = 1,279;

Number of cells analyzed in the PFC:

WT, *Baz1a*+ = 493, *Baz1a*− = 1521; Mut, *Baz1a*+ = 280, *Baz1a*− = 1,683

Number of cells analyzed in the SC:

WT, *Baz1a*+ = 321, *Baz1a*− = 1,459; Mut, *Baz1a*+ = 191, *Baz1a*− = 1641

Figure 6 (D)

Neurons categorized into three groups based on normalized c-fos intensity;

bottom 33%, *n* = 47 neurons; middle 33%, *n* = 65 neurons; top 33%, *n* = 56 neurons.

Figure 6 (E)

Neurons categorized into three groups according to the LFC index;

bottom 33%, *n* = 832 neurons; middle 33%, *n* = 830 neurons; top 33%, *n* = 831 neurons.

Figure 6 (H)

Number of neurons analyzed for dendritic arborization;

WT c-fos (+), n = 7; WT c-fos (−), n = 12

Mut c-fos (+), n = 10; Mut c-fos (−), n = 11

Figure 6 (J)

Number of dendrites analyzed for spine density:

WT c-fos (+), *n* = 51 from 4 animals

WT c-fos (−), *n* = 69 from 5 animals

Mut c-fos (+), *n* = 69 from 4 animals

Mut c-fos (−), *n* = 58 from 4 animals

Figure 6 (K)

Correlation between the mean spine density and c-fos/GFP intensity ratio;

WT: *n* = 25 neurons from 5 animals

Mut: *n* = 31 neurons from 4 animals

Figure 6 (M)

Size distribution of putative peri-somatic inhibitory synapses; number of puncta:

c-fos (+): WT, *n* = 111; Mut, *n* = 157

c-fos (−): WT, *n* = 193; Mut, *n* = 194

Figure 6 (N)

Mean area size and number of GAD65-positive putative synapses;

Number of puncta: same as in Figure 6 (M),

Number of cells:

c-fos (+): WT, *n* = 8; Mut, *n* = 12

c-fos (−): WT, *n* = 15; Mut, *n* = 15

Figure 7 (C and D)

Feedforward and feedback signals between A1 and A2;

n = 20 sessions from 5 WT mice and 16 sessions from 4 Mut mice.
